# LC-QTOF-MS/MS Based Molecular Networking Approach for the Isolation of α-Glucosidase Inhibitors and Virucidal Agents from *Coccinia grandis* (L.) Voigt

**DOI:** 10.3390/foods10123041

**Published:** 2021-12-07

**Authors:** Maharani A. Astiti, Akanitt Jittmittraphap, Pornsawan Leaungwutiwong, Nopporn Chutiwitoonchai, Patcharee Pripdeevech, Chulabhorn Mahidol, Somsak Ruchirawat, Prasat Kittakoop

**Affiliations:** 1 Chulabhorn Graduate Institute, Program in Chemical Sciences, Chulabhorn Royal Academy, Laksi, Bangkok 10210, Thailand; maharaniajengastiti@gmail.com (M.A.A.); mahidol_natlab@cri.or.th (C.M.); somsak@cri.or.th (S.R.); 2 Department of Microbiology and Immunology, Faculty of Tropical Medicine, Mahidol University, 420/6 Ratchawithi Rd., Ratchadewee, Bangkok 10400, Thailand; akanitt.jit@mahidol.ac.th (A.J.); pornsawan.lea@mahidol.ac.th (P.L.); 3 Virology and Cell Technology Research Team, National Center for Genetic Engineering and Biotechnology (BIOTEC), National Science and Technology Development Agency (NSTDA), 113 Thailand Science Park, Phahonyothin Rd., Khlong Nueng, Khlong Luang, Pathumthani 12120, Thailand; nopporn.chu@biotec.or.th; 4 School of Science, Mae Fah Luang University, Muang, Chiang Rai 57100, Thailand; patcharee.pri@mfu.ac.th; 5 Chulabhorn Research Institute, Kamphaeng Phet 6 Road, Laksi, Bangkok 10210, Thailand; 6 Center of Excellence on Environmental Health and Toxicology (EHT), CHE, Ministry of Education, Bangkok 10210, Thailand

**Keywords:** flavonoid glycosides, diabetes mellitus, antidiabetic vegetable, influenza A virus H1N1, GNPS molecular networking, LC-MS analysis, apiose-containing glycosides

## Abstract

*Coccinia grandis* or ivy gourd is an edible plant. Its leaves and fruits are used as vegetable in many countries. Many works on antidiabetic activity of a crude extract of *C*. *grandis*, i.e., in vitro, in vivo, and clinical trials studies, have been reported. Profiles of the antidiabetic compounds were previously proposed by using LC-MS or GC-MS. However, the compounds responsible for antidiabetic activity have rarely been isolated and characterized by analysis of 1D and 2D NMR data. In the present work, UHPLC-ESI-QTOF-MS/MS analysis and GNPS molecular networking were used to guide the isolation of α-glucosidase inhibitors from an extract of *C*. *grandis* leaves. Seven flavonoid glycosides including rutin (**1**), kaempferol 3-*O*-rutinoside (**2**) or nicotiflorin, kaempferol 3-*O*-robinobioside (**3**), quercetin 3-*O*-robinobioside (**4**), quercetin 3-*O*-β-D-apiofuranosyl-(1→2)-[α-L-rhamnopyranosyl-(1→6)]-β-D-glucopyranoside (**5**) or CTN-986, kaempferol 3-*O*-β-D-api-furanosyl-(1→2)-[α-L-rhamnopyranosyl-(1→6)]-β-D-glucopyranoside (**6**), and kaempferol 3-*O*-β-D-apiofuranosyl-(1→2)-[*α*-L-rhamnopyranosyl-(1→6)]-β-D-galactopyranoside (**7**) were isolated from *C*. *grandis* leaves. This is the first report of glycosides containing apiose sugar in the genus *Coccinia*. These glycosides exhibited remarkable α-glucosidase inhibitory activity, being 4.4–10.3 times more potent than acarbose. Moreover, they also displayed virucidal activity against influenza A virus H1N1, as revealed by the ASTM E1053-20 method.

## 1. Introduction

*Coccinia grandis* (L.) Voigt or ivy gourd is an edible plant under the family of *Cucurbitaceae*. It is a perennial climber with the synonym of *Coccinia indica* Wight & Arn. Ivy gourd, *C*. *grandis*, is native to many regions of tropical and subtropical Asia, America, and the Pacific. In Thailand, ivy gourd is called “Tum Lueng” and used as a common vegetable in Thai cuisine. *C*. *grandis* is also used as vegetable in many countries, for example, India, Sri Lanka, Bangladesh, Indonesia, Philippines, Cambodia, Vietnam, Malaysia, and Myanmar. In Indonesia and Sri Lanka, leaves of *C*. *grandis* are used to treat diabetes [1]. Traditional use of *C*. *grandis* as a wound dressing is known, and compounds responsible for wound healing were identified by LC-ESI-MS/MS analysis [2]. *C*. *grandis* is one of the herbal remedies to treat jaundice, skin eruption, cough, diabetes, and liver weakness [3]. *C*. *grandis* is used to treat fever, herpes zoster, infectious wound, and eye inflammation in Thailand. Since *C*. *grandis* is used in many traditional medicines, its crude extracts were intensively studied for biological activities, for example, anti-inflammatory and anti-apoptotic effect [4], and attenuation of monosodium glutamate mixed high-lipid diet induced systemic damage in rats [5]. Crude leaf extract of *C*. *grandis* contained serine protease inhibitors, which could induce protective immune responses in murine visceral leishmaniasis [6]. Crude extract of *C*. *grandis* leaves could inhibit NF-kB and caspase 3 mediated signaling in a rat model [7]. The extract of *C*. *grandis* with the collagen coated nanofibrous scaffold was found to accelerate the healing process of the wound [8], and recent work revealed that leaf extract of *C*. *grandis* was involved in wound-healing process by reducing oxidative stress injury with potential use in skincare products [9]. Crude extract of *C*. *grandis* displayed starch hydrolase inhibitory activity, and this plant was beneficial as functional food [10]. Polyprenol isolated from *C*. *grandis* was identified as the active compound for antidyslipidemic activity in high-fat diet-fed hamster model [11]. Crude extract of *C*. *grandis* was found to prevent of diabetic nephropathy through inhibition of glycation and toxicity to erythrocytes and HEK293 cells [12], while its root extract inhibited adipocyte differentiation and decreased mRNA levels of adipogenic genes, thus preventing obesity [13]. Fruit extract of *C*. *grandis* showed aldose reductase inhibitory and antioxidant activities and may be useful for prevention and management of diabetes [14].

**Figure 1:**
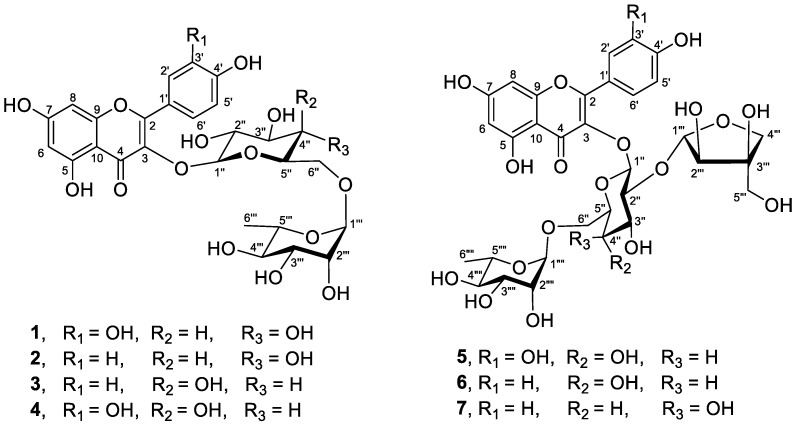
Structures of compounds **1**–**7** isolated from leaves of *C*. *grandis*.

Extracts of *C. grandis* are prevalent for antidiabetic property, for example, inducing GLUT4 translocation, accompanied by an increase of intracellular glucose concentrations [15] and prevention of diabetic complications [16]. Recent clinical trial revealed that patients with newly diagnosed type 2 diabetes mellitus treated with *C. grandis* considerably improved their glycemic and lipid profile parameters with well-tolerated safety [1]. While a number of works reported biological activities of crude extracts of *C. grandis*, isolation and characterization of chemical constituents in this plant have rarely been investigated. Most investigations of the compounds in *C. grandis* employed LC-MS or GC-MS analysis, for example, LC-ESI MS/MS analysis for the identification of compounds responsible for wound healing and anti-bacterial activity [2], UPLC-QTOF-MS analysis for metabolite profiling of the bioactive fraction toward α-glucosidase inhibitory activity [17], and GC-MS analysis of methanol and water extracts of *C. grandis* [14]. LC-MS and GC-MS techniques can propose only tentative structures or the tentative identity of compounds, while analysis of NMR and MS data of the isolated pure compounds provides the precise structures of bioactive natural products. As mentioned above, many works have shown antidiabetic activity of a crude extract *C. grandis*, i.e., in vitro, in vivo, and clinical trial studies; however, the active compounds in this plant have rarely been isolated and characterized by analysis of NMR data. We also found that a crude leaf extract of *C. grandis* displayed α-glucosidase inhibitory activity, one of the targets for antidiabetic drugs. Herein, we report the isolation and characterization of α-glucosidase inhibitors in *C. grandis*, which is guided by Global Natural Product Social (GNPS) molecular networking generated from LC-MS/MS analysis. Seven flavonoid glycosides, **1**–**7**, were isolated from a leaf extract of *C. grandis* (Figure 1), and they were evaluated for α-glucosidase inhibitory and virucidal activities.

## 2. Materials and Methods

### 2.1. Chemicals

Standard compounds for LC-MS including rutin, kaempferol, and quercetin were purchased from TCI Chemicals (Tokyo, Japan). *p*-Nitrophenyl-α-D-glucopyranoside (*p*-NPG, ≥98%) and acarbose were from TCI Chemicals (Tokyo, Japan). α-Glucosidase enzyme (*Saccharomyces cerevisiae,* lyophilized powder, 10 U/mg) were purchased from Sigma Aldrich (St. Louis, MO, USA). Dimethylsulfoxide (DMSO) was purchased from RCI Labscan (Bangkok, Thailand).

### 2.2. Plant Materials and Extraction Method

Leaves of *Coccinia grandis* (L.) Voigt were collected from Pathum Thani Province, Thailand. Fresh leaves of *C*. *grandis* (0.89 kg) were chopped into small pieces using an electric blender. It was extracted with methanol at room temperature (3 × 2.5 L; each by maceration in methanol for 3 days). A methanol extract was evaporated using a rotary evaporator to give 27.86 g of a crude extract.

### 2.3. UHPLC-ESI-QTOF-MS/MS Analysis

A crude extract of *C*. *grandis* leaves was analyzed by UHPLC Agilent 1290 infinity II coupled to the Agilent 6545 LC-QTOF/MS system. A crude extract (1 mg) or the standard compound was dissolved in 1 mL of methanol to make stock solution at concentrations of 1 mg/mL. A total of 100 µL of stock solution was diluted with 900 µL of methanol to obtain the final concentration of 100 µg/mL. The solution then was filtered through a 0.22 µm filter and transferred into a 2 mL LC vial. The HPLC column was Waters XBridge C_18_ (2.1 × 100 mm, 2.5 µm), and at the flow rate was at 0.4 mL/min with an injection volume of 1 µL. Mobile phase consisted of 0.1% of formic acid in water (A) and 0.1% of formic acid in acetonitrile (B) The gradient elution was applied using the following conditions: 0–13 min, 5–17% B; 13–20 min, 17–100% B; 20–25 min, 100% B; and 25–27 min, 100–5%. A post-run of 6 min was set to equilibrate the column between analyses.

Dual AJS (Agilent Jet Stream) ESI was used as an interface for LC-MS system, which was arranged with specific parameters: sheath gas flow rate, 12 L/min; sheath gas temperature, 250 °C; nebulizer pressure, 45 psig; gas flow rate, 11 L/min; gas temperature, 300 °C. LC-MS was operated in full-scan mode, performing positive and negative ionizations, with a scan range from 50–1050 *m/z*, and the scan rate was 1 spectra per second. Auto-MS^2^ was performed using fixed collision energies of 10, 20, and 40 eV. The scan rate was set at three spectra per second with MS/MS scan range from 50–1100 *m/z*. Isolation width MS/MS was set at ±4 *m/z*, and the maximum precursor was three per cycle. The reference solutions were prepared to provide internal reference masses for mass correction in positive and negative modes of operation. The calibrant masses at *m/z* 121.0509 (purine) and *m/z* 992.0098 (HP-0921) were captured as standard ion peaks in positive mode, while the ions at *m/z* 112.9855 (TFA anion) and *m/z* 1033.9881 (HP-0921 + TFA anion) were captured in the negative mode.

### 2.4. MS-Based Molecular Networking

Prior to the generation of molecular networking, MSconvert, a free software from ProteoWizard was used to convert MS/MS data into an open file format (mzXML). The mzXML files were subjected to the GNPS (Global Natural Product Social Molecular Networking) website to create molecular networks [18]. Several parameters in MSconvert were set up, i.e., 32-bit of binary encoding precision, peak picking, and MS-Levels 1–2 were selected, while zlib compression was unchecked [19]. FTP client, WinSCP, was employed to upload all mzXML MS/MS data format using the host name ccms-ftp01.ucsd.edu. (port 21). All these data were available in the GNPS website once uploaded and ready to create the molecular networks on GNPS website (https://gnps.ucsd.edu/ProteoSAFe/static/gnps-splash.jsp, accessed on 15 August 2021) [20]. The GNPS networking parameters under the “basic option” section was arranged by keeping the precursor ion and fragment ion mass tolerance at 0.5 and 0.02 Da, respectively. This setting allows the algorithm to generate a single consensus spectrum within specific range. To produce the molecular network and lower the network complexity, the matched fragment ion was kept to a minimum of 4 (MS fragments) and the minimum cosine similarity score for connecting one node to another was set to 0.7. The TopK value was set to 10 for maintaining the edge between two nodes in a network. The cosine score is ranged between 0.7–1, where a cosine score of 1 denotes high similarity. The TopK value of 10 defines as the maximum number of nodes, which is connected to a single node. To evaluate the quality of annotation of the corresponding cosine score, several parameters such as the total of fragment ions, peak intensities, and parent mass accuracy are taken into consideration [19]. The molecular networks generated from the GNPS system were imported into Cytoscape 3.8.2 to allow more approachable visual exploration of the detectable metabolite profile in a crude extract and the correlation of standard compounds with their analogs [19].

### 2.5. NMR Experiments

NMR spectra were recorded on a Bruker AVANCE 400 MHz spectrometer (400 MHz for ^1^H NMR and 100 MHz for ^13^C NMR) at 24 °C (297 K). The samples were dissolved in 500 μL of methanol-*d*_4_ using a 5.0 mm Wilmad-LabGlass™ NMR tube. The residual solvent signals, methanol-*d*_4_ at δ_H_ 3.31/δ_C_ 49.0 ppm and tetramethylsilane (TMS), were served as the internal standard.

### 2.6. Isolation of Flavonoid Glycosides ***1**–**7***

A portion (10 g) of a crude extract of *C. grandis* leaves was fractionated by Sephadex LH-20 column chromatography (4 × 78 cm), which was eluted with methanol, giving twenty-two fractions (F1–F22). Based on analysis of ^1^H NMR spectra, fractions F9, F10, F11, F12, F13, and F14 had NMR signals of flavonoid glycosides, and these fractions were further purified using semi-preparative C_18_ HPLC column (SunFire 5.0 µm, 19 × 250 mm). An isocratic elution with acetonitrile: water (20:80) was applied for HPLC, and a flow rate was at 10 mL/min with the total run time of 30 min. HPLC separation of the fractions F10–F13 gave three major compounds, i.e., rutin (**1**, t_R_ 14 min, 137.8 mg), kaempferol 3-*O*-rutinoside (**2**, t_R_ 21 min, 74.7 mg), and kaempferol 3-*O*-robinobioside (**3**, t_R_ 18.4 min, 23.5 mg). HPLC separation of the fraction F14 gave a mixture of rutin and quercetin 3-*O*-robinobioside (**1** and **4**, t_R_ 13.5 min, 22.6 mg), while HPLC separation of the fractions F9 and F10 provided three compounds including quercetin 3-*O*-β-D-apiofuranosyl-(1→2)-[α-L-rhamnopyranosyl-(1→6)]-β-D-glucopyranoside (**5**, t_R_ 9 min, 14.1 mg), kaempferol 3-*O*-β-D-apiofuranosyl-(1→2)-[α-L-rhamnopyranosyl-(1→6)]-β-D-glucopyranoside (**6**, t_R_ 12 min, 8.3 mg), and kaempferol 3-*O*-β-D-apiofuranosyl-(1→2)-[α-L-rhamnopyranosyl-(1→6)]-β-D-galactopyranoside (**7**, t_R_ 11.5 min, 1.5 mg).

### 2.7. In-Vitro α-Glucosidase Inhibitory Assay

The α-glucosidase assay was performed as described by Liu et al. [21] with slight modification. The reaction mixture consisting of 50 µL of potassium phosphate buffer (PBS, 0.1 M, pH 6.8) and 20 µL of α-glucosidase (0.5 U/mL) was pre-incubated with 20 µL of the test sample at 37 °C for 15 min in a 96-well plate. The mixture was added 20 µL *p-*NPG (1 mM) and incubated again at 37 °C for 15 min. A total of 50 µL of Na_2_CO_3_ (0.2 M) was added to terminate the enzymatic activity. The absorbance value of each well was measured immediately using a microplate reader at 405 nm. Acarbose was used as a positive control. All glycosides were dissolved in 50% DMSO, while a crude extract was dissolved in 100% DMSO. The experiment was performed in triplicate. The enzyme inhibition rate was calculated as follows:

(1) $$\left[1-\frac{\left(OD_{sample}-OD_{sample\;blank}\right)}{\left(OD_{control}-OD_{blank}\right)}\right]\;\times \;100$$

where *OD_sample_* represents the absorbance of PBS + enzyme + sample + *p-*NPG; *OD_sample blank_* represents the absorbance of PBS + sample; *OD_control_* represents the absorbance of PBS + enzyme + solvent + *p-*NPG; *OD_blank_* represents the absorbance of PBS + solvent. The inhibitory activity is expressed as IC_50_ (half minimal inhibitory concentration) value. Data were normalized, and the IC_50_ values were calculated using GraphPad Prism 9.2.0.

### 2.8. Evaluation of Virucidal Activity

Virucidal activity of glycosides was evaluated against influenza A virus H1N1 by the American Society for Testing and Materials (ASTM) method No. ASTM E1053-20 [22]. Prior to the virucidal assay, cytotoxicity of glycosides in Madin-Darby Canine Kidney (MDCK) cells was preliminarily examined at the concentrations of 500, 250.0, 125.0, 62.5, 31.3, 15.6, and 7.80 µg/mL by the MTT assay. The concentrations at which the compounds show more than 70% cell viability are considered non-toxic concentrations. Glycosides were tested for virucidal activity at their non-toxic concentrations. Influenza A virus H1N1 was cultured on MDCK host cells. The virus was exposed to glycosides for 10 min, and then it was neutralized and filtered. The filtrate was subjected to a serial 10-fold virus dilutions and applied in quadruplicate to 96-well plates containing MDCK monolayer. Cells in an individual well were observed for cytopathic effects. Amounts of infectious virus were quantified by the Spearman–Karber method. A total of 0.21% Sodium hypochlorite was used as a positive virucidal agent, which has a Log reduction of >4.4, accounting for 99.99% of the reduction in the virus [23].

## 3. Results and Discussion

### 3.1. Profile of Compounds in a Crude Extract of C. Grandis Revealed by ^1^H NMR Spectrum and LC-MS/MS Analysis, and GNPS Molecular Networking of Flavonoid Glycosides

^1^H NMR spectrum (in CD_3_OD) of a crude extract of *C***.***grandis* leaves showed signals of flavonoid at the chemical shifts (δ_H_) 6–8 ppm, which belong to flavonoids, kaempferol, and quercetin (Supplementary Figure S1). LC-MS analysis of a crude extract of *C. grandis* was performed, and its TIC chromatogram is shown in Supplementary Figure S2. LC-MS/MS analysis showed 32 tentatively identified compounds in a methanolic extract of *C. grandis* obtained from LC-MS/MS analysis (Table 1). Among them, four flavonoid glycosides including rutin (**1**), kaempferol 3-*O*-rutinoside or nicotiflorin (**2**), quercetin 3-*O*-β-D-apiofuranosyl-(1→2)-[α-L-rhamnopyranosyl-(1→6)]-β-D-glucopyranoside or CTN-986 (**5**), and kaempferol 3-*O*-β-D-apiofuranosyl-(1→2)-[α-L-rhamnopyranosyl-(1→6)]-β-D-galactopyranoside (**7**) were detected by LC-MS/MS analysis at the retention times (RT) of 9.387, 7.086, 8.867, and 9.660 min, respectively (Table 1). In addition, we also performed analysis of the crude extract using LC-MS/MS molecular networking approach by employing the GNPS system to create molecular networks. All converted MS/MS data were used to generate network nodes in which corresponding to a consensus MS/MS spectrum having similar precursor mass. The network nodes were connected via edges based on the cosine similarity score where the high score reflects the close relatedness between two nodes.

**Figure 2:**
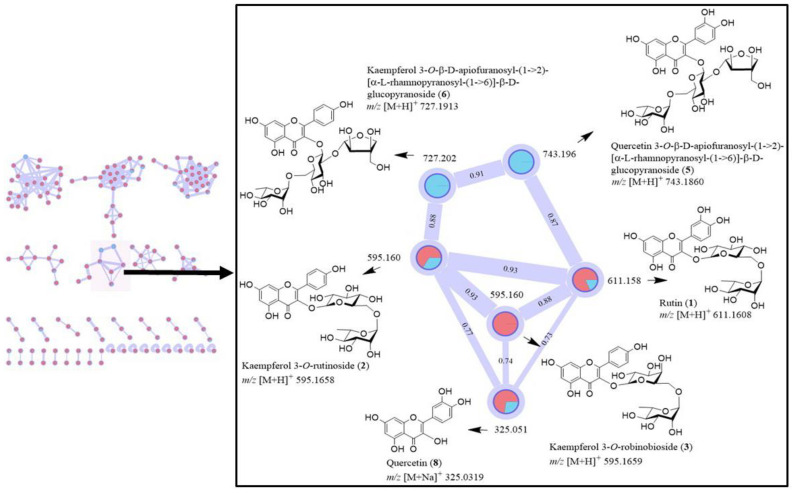
Molecular networking of flavonoid glycosides in a crude leaf extract of *C*. *grandis*. Two colors of node, red and blue, represent the standard compounds and a crude extract, respectively.

The GNPS molecular networking was performed for a crude leaf extract of *C*. *grandis* using a positive ion mode (Supplementary Materials, Figure S3). Initially, a commercially available quercetin (**8**) was used as a standard compound for the GNPS molecular networking of a crude leaf extract, leading to the networking generation shown in Figure 2. Quercetin (**8**) had the networking with flavonoids **1**, **2**, **5**, and **6** (Figure 2). We then performed the isolation of *C*. *grandis* extract, and obtained compounds **1**–**3** as major constituents. Subsequently, we used flavonoids **1**–**3** as standard compounds for the GNPS molecular networking, and the networking is depicted in Figure 2. Red nodes in Figure 2 are of standard compounds including rutin (**1**), kaempferol 3-*O*-rutinoside (**2**), kaempferol 3-*O*-robinobioside (**3**), and quercetin (**8**), while blue nodes represent compounds in a crude extract. The networking revealed a few related compounds in a crude extract (Figure 2). Kaempferol 3-*O*-robinobioside (**3**) (*m/z* 595.1659, Figure 2) showed relation to a few nodes, i.e., the ions at *m/z* 611.1608 [M + H]^+^, 595.1658 [M + H]^+^, and 325.0319 [M + Na]^+^, which are rutin (**1**), kaempferol 3-*O*-rutinoside (**2**), and quercetin (**8**), respectively. They have cosine similarity scores of 0.75-0.93 (Figure 2). Furthermore, rutin (**1**) with the *m/z* 611.1608 [M + H]^+^ showed the connection to the node at *m/z* 743.1860 [M + H]^+^ of quercetin 3-*O*-β-D-apiofuranosyl-(1→2)-[α-L-rhamnopyranosyl-(1→6)]-β-D-glucopyranoside (**5**). The precursor ion of kaempferol 3-*O*-rutinoside (**2**) (*m/z* 595.1658 [M + H]^+^) was found to be kaempferol 3-*O*-β-D-apiofuranosyl-(1→2)-[α-L-rhamnopyranosyl-(1→6)]-β-D-glucopyranoside (**6**) (*m/z* 727.1913 [M + H]^+^). In addition to the information of tentatively identified compounds obtained from LC-MS/MS analysis shown in Table 1, the GNPS molecular networking provides further information on the presence of some flavonoid glycosides, and it confirms the existence of flavonoids in a crude extract by cosine score. The information from this molecular networking (Figure 2) led to the isolation of compounds **1**, **2**, **3**, **5**, and **6**, whose structures were characterized by analysis of NMR data. It is worth mentioning that there are a number of clusters of the GNPS molecular networking (Supplementary Figure S3). However, other clusters could not be analyzed because there were no authentic standard compounds available. Moreover, ^1^H NMR spectrum indicated the presence of flavonoids as major compounds in *C*. *grandis* leaf extract; with this information, we focused only on the networking of flavonoids.

### 3.2. Isolation and Characterization of Flavonoid Glycosides

Flavonoids are known to have α-glucosidase inhibitory, antimicrobial, and antiviral activities [24,25,26], and they are also used as natural additives [27]. As ^1^H NMR spectrum and GNPS molecular networking suggested the presence of flavonoids in an extract of *C*. *grandis*, isolation of flavonoids was performed, giving seven flavonoid glycosides **1**–**7** (Figure 1). ^1^H, ^13^C NMR, and MS spectra of compounds **1**–**7** are in the Supplementary Figure S4–S26. Rutin (**1**), a yellow amorphous powder, had the molecular formula of C_27_H_30_O_16_ as deduced from high-resolution electrospray ionization mass spectrometry (HRESI-MS). The structure of rutin (**1**) was confirmed by analysis of 1D and 2D NMR spectra, whose data were identical to those reported in the literature [28]. Kaempferol 3-*O*-rutinoside (**2**) or nicotiflorin was obtained as yellow amorphous solid with a molecular formula of C_27_H_30_O_15_ (from HRESI-MS). Analysis of NMR spectra established the structure of glycoside **2**, and their NMR data were in good agreement with published values [29]. Kaempferol 3-*O*-robinobioside (**3**) or kaempferol 3-*O*-alpha-L-rhamnopyranosyl-(1→6)-beta-D-galactopyranoside was obtained as yellow amorphous solid with the molecular formula of C_27_H_30_O_15_ as inferred from HRESI-MS. NMR data of glycoside **3** were in good agreement with those reported in the literature [30]. Quercetin 3-*O*-robinobioside (**4**) had a molecular formula of C_27_H_30_O_16_ as deduced from HRESI-MS. Hassan and co-workers first reported the structure of glycoside **4**; however, they obtained glycoside **4** as a mixture with rutin (**1**) [31]. In this work, we also obtained the same mixture of glycosides **4** and **1** with the proportion ratio of 1.00:0.79. On the reversed phase HPLC chromatogram, a mixture of **4** and **1** exhibited only a single peak, suggesting that it is an inseparable mixture. It is worth mentioning that the first sugar unit attached to a flavonoid skeleton is different among glycosides **1**–**4**; compounds **1** and **2** have glucose, while compounds **3** and **4** have galactose (Figure 1). Glycoside **5** was obtained as yellow amorphous solid with the molecular formula of C_32_H_38_O_20_ (by HRESI-MS). The structure of **5** was established by analysis of spectroscopic data, and it was identified as quercetin 3-*O*-β-D-apiofuranosyl-(1→2)-[α-L-rhamnopyranosyl-(1→6)]-β-D-glucopyranoside or CTN-986 or apiorutin, whose NMR data were identical to those of published values [31,32,33]. Glycoside **6** was obtained as yellow amorphous solid with the molecular formula of C_32_H_38_O_19_ (by HRESI-MS), and it was identified as kaempferol 3-*O*-β-D-apiofuranosyl-(1→2)-[α-L-rhamnopyranosyl-(1→6)]-β-D-glucopyranoside. Spectroscopic data of **6** were identical to those reported in the literature [32]. Glycoside **7** was obtained as yellow amorphous solid, and it had the same molecular formula of C_32_H_38_O_19_ (by HRESI-MS) as that of compound **6**. Compound **7** was identified as kaempferol 3-*O*-β-D-apiofuranosyl-(1→2)-[α-L-rhamnopyranosyl-(1→6)]-β-D-galactopyranoside, and its NMR data were in good agreement with those reported in the literature [33]. NMR data of glycosides **1**–**7** are in the Supplementary Tables S1 and S2.

Recently, rutin (**1**) and kaempferol 3-*O*-rutinoside (**2**) were reported to be the constituents in *C*. *grandis* by LC-ESI-MS/MS analysis [2,17]. It is worth mentioning that kaempferol 3-*O*-rutinoside (**2**) has the same molecular weight as that of kaempferol 3-*O*-robinobioside (**3**). Compound **2** has glucose, but glycoside **3** has galactose, and both sugars has the same molecular weight; therefore, LC-MS/MS technique hardly distinguishes between the glycosides **2** and **3**. For this reason, when analyzing by LC-MS/MS, only glycosides **1** and **2** with glucose in the molecules were proposed to be the constituents in *C*. *grandis* [2,17]. Again, glycoside **6** has the same molecular weight as that of compound **7**, and the difference is the presence of glucose in glycoside **6** and galactose in **7**. Therefore, the LC-MS/MS method hardly distinguishes between compounds **6** and **7**. The present work is the first report on the isolation and characterization of glycosides **1**–**7** from *C*. *grandis*. Moreover, this work reveals that there are two closely related sugars, glucose or galactose, attached to the position 3 of a flavonoid backbone, thus generating the chemical diversity in *C*. *grandis*. Increase in chemical diversity of flavonoid glycosides would lead to their diverse biological activities. Unexpectedly, the glycosides with a sugar apiose, i.e., compounds **5**–**7**, were isolated from a crude extract of *C*. *grandis* leaves. Among them, 3-*O*-β-D-apiofuranosyl-(1→2)-[α-L-rhamnopyranosyl-(1→6)]-β-D-glucopyranoside (**5**), also known as CTN-986, previously isolated from cottonseeds, was found to exhibit antidepressant and cytoprotective effects [34]; it could enhance the proliferation of hippocampal neural progenitor cells, possibly via the 5-HT1A receptor [35]. A study on pharmacokinetics and tissue distribution revealed that CTN986 could be absorbed and distributed into tissues in the mice model [36,37]. Glycosides with a sugar apiose have diverse biological activities, and the roles of apiosylated compounds in plants are considered a challenging open research area [38]. Therefore, apiose-containing compounds have recently been received attention, both in biosynthesis and chemical synthesis [39,40].

### 3.3. α-Glucosidase Inhibitory Activity of the Isolated Flavonoid Glycosides

**Figure 3:**
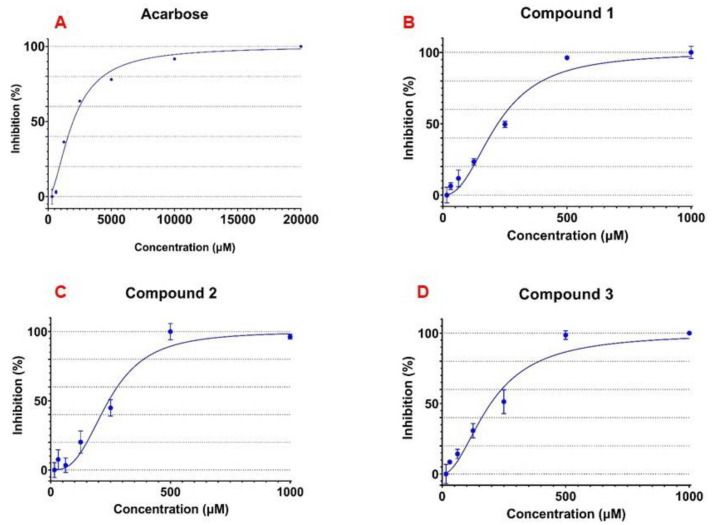

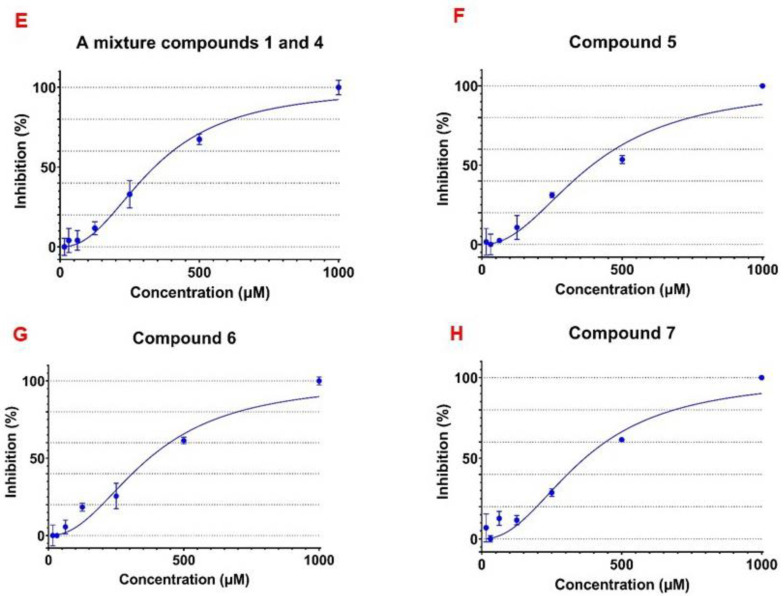
Dose dependent α-glucosidase inhibitory activity for acarbose (**A**), compound **1** (**B**), compound **2** (**C**), compound **3** (**D**), a mixture of compound **4** and **1** (**E**), compound **5** (**F**), compound **6** (**G**), and compound **7** (**H**).

There have been a number of reports on the antidiabetic activity of *C*. *grandis* crude extracts, as mentioned earlier in the introduction. However, the compounds responsible for the antidiabetic activity have rarely been isolated and characterized. In the present work, we also found that a crude extract of *C*. *grandis* leaves exhibited α-glucosidase inhibitory activity with IC_50_ value of 1.24 ± 0.07 mg/mL (Supplementary Figure S27). Seven glycosides **1**–**7** isolated from *C*. *grandis* leaves were evaluated for α-glucosidase inhibitory activity (Table 2). Acarbose, the α-glucosidase inhibitor drug, was used as a standard compound. Curves for dose-dependent α-glucosidase inhibitory activity of acarbose and glycosides are in Figure 3. As shown in Table 2, rutin (**1**), kaempferol 3-*O*-rutinoside (**2**), kaempferol 3-*O*-robinobioside (**3**), a mixture of quercetin 3-*O*-robinobioside (**4**) and rutin (**1**), quercetin 3-*O*-β-D-apiofuranosyl-(1→2)-[α-L-rhamnopyranosyl-(1→6)]-β-D-glucopyranoside (**5**), kaempferol 3-*O*-β-D-apiofuranosyl-(1→2)-[α-L-rhamnopyranosyl-(1→6)]-β-D-glucopyranoside (**6**), and kaempferol 3-*O*-β-D-apiofuranosyl-(1→2)-[α-L-rhamnopyranosyl-(1→6)]-β-D-galactopyranoside (**7**) exhibited α-glucosidase inhibitory activity with IC_50_ values of 243.4, 235.8, 195.4, 342.2, 431.0, 455.6, and 432.5 µM, respectively. These glycosides are 4.4–10.3 times more potent than acarbose (IC_50_ value of 2023.3 µM). The most potent α-glucosidase inhibitor was kaempferol 3-*O*-robinobioside (**3**), exhibiting an IC_50_ value of 195.4 µM, 10.3 times more potent than the acarbose drug (Table 2). It is worth mentioning that the activity (IC_50_ of 195.4 µM) of glycoside **3** with galactose was slightly more active than that (IC_50_ of 235.8 µM) of its corresponding isomer **2** attached with glucose (Table 2). Among glycosides **1**–**7**, rutin (**1**) and kaempferol 3-*O*-rutinoside (**2**) isolated form leaves of *Morus atropurpurea* were previously reported to have α-glucosidase inhibitory activity [41]. Compounds **3**–**7** have never been reported as α-glucosidase inhibitors so far. The present work characterizes the structures of α-glucosidase inhibitors in *C*. *grandis* for the first time. However, there may be other classes of compounds in this plant acting as α-glucosidase inhibitors. All compounds isolated from *C*. *grandis* leaves exhibited better α-glucosidase inhibitory activity than acarbose, and this work supports the recent clinical trial on *C*. *grandis* leaves for the treatment of type 2 diabetes patients [1].

### 3.4. Virucidal Activity of the Isolated Flavonoid Glycosides

Six flavonoid glycosides **1**–**6** were evaluated for virucidal activity against influenza A virus H1N1 (Table 3). A small amount of compound **7** was obtained, which was not sufficient for virucidal activity testing. Compounds **1**–**3** had cell viability more than 70% at the concentrations of 125.0 µg/mL or at lower concentrations, while a mixture of **4** and **1**, compounds **5**–**6,** had cell viability of more than 70% at the concentrations of 500.0 µg/mL or at lower concentrations (Table 3). Virucidal activity of the isolated glycosides were reported at the concentrations with cell viability more than 70%. As shown in Table 3, at the concentration of 125.0 µg/mL, glycosides **1**–**3** showed virucidal activity with Log reduction of 1.80–1.95, accounting for 99% reduction of virus. Compound **3** at lower concentrations of 31.3 and 15.6 µg/mL retained its virucidal potency with Log reduction around 1.9 that could reduce 99% of virus. A mixture of **4** and **1**, compounds **5** and **6,** at the concentrations of 500.0 and 250.0 µg/mL exhibited virucidal activity with Log reduction of 1.93–2.41, accounting for a 99% reduction of virus, and these glycosides could retain virucidal potency with the Log reductions of 1.8–1.9 at lower concentrations, even at the lowest concentration tested at 15.6 µg/mL (Table 3). Overall, flavonoid glycosides in leaves of *C*. *grandis* exhibited moderate virucidal activity with 99% reduction of virus, when compared to a positive virucidal agent, 0.21% sodium hypochlorite, which is a common household disinfectant that could reduce 99.99% of virus (Log reduction of >4.4). Rutin (**1**) was previously reported to act as a potent hepatitis C virus entry inhibitor [42], and recently, an in silico study revealed that it could bind with the SARS-CoV-2 (COVID-19) main protease [43]. Kempferol 3-*O*-rutinoside (**2**) was reported to possess strong inhibition towards dengue virus serine protease [44]. Glycosides **1**–**3** were reported to exhibit potent antiviral activity against herpes simplex virus-1 and -2 [45]. Recently, flavanone glycosides from *Dracocephalum* spp. were reported to exhibit virucidal activity against feline calicivirus [46]. The present work underscores the importance of glycosides as sources of natural antiviral and virucidal agents.

## 4. Conclusions

In the present study, GNPS molecular networking was used to guide chemical investigation of compounds in a crude extract of *C. grandis* leaves. Seven flavonoid glycosides **1**–**7** were isolated from *C. grandis* extract, and they were found to be potent α-glucosidase inhibitors, which were even more potent than acarbose, an antidiabetic drug. This is the first report on the systematic isolation and identification of α-glucosidase inhibitors in *C*. *grandis* and on the α-glucosidase inhibitory activity of flavonoid glycosides **3**–**7**. The finding of potent α-glucosidase inhibitors in *C. grandis* leaves supports the recent clinical trial on the use of *C. grandis* leaves for the treatment of patients with type 2 diabetes mellitus, and it revealed that *C. grandis* remarkably improved a glycemic profile of patients [1]. Since *C. grandis* leaves are used as vegetables in many countries, they may partly contribute the benefits for people with type 2 diabetes mellitus. Moreover, glycosides **1**–**6** were also found to have moderate virucidal activity against influenza A virus H1N1. Foodborne viruses, for example hepatitis A and hepatitis E viruses, have caused problems in the food industry, thus causing a serious problem for public health [47]. Inactivation of foodborne viruses by natural products is one of the approaches for the control of these viruses [48,49]. This work provides the information about virucidal flavonoid glycosides in *C*. *grandis*, which is used as a common food ingredient in many Asian dishes.

## Figures and Tables

**Table 1:** Tentatively identified compounds in a methanolic extract of *C*. *grandis* leaves obtained from LC-MS/MS analysis. Compounds were identified by Metlin Database (M), or Human Metabolome Database (H) or isolation and characterization (Isolated).

No.	RT (Min)	Compounds (Identification by Database or Isolation)	Molecular Formula	Mass	Adduct Ions	Observed *m/z*	Calculated *m/z*	Δ (ppm)	Fragment Ions (*m/z*)
**1**	0.638	Maltopentaose (M)	C_30_H_52_O_26_	828.2741	(M − H)−	827.2674	827.2674	0.71	683.2247, 533.1721, 471.0754, 387.1144, 341.1092, 299.0983, 191.0562, 162.8391, 133.0143
**2**	0.640	L-Mannitol (H, M)	C_6_H_14_O_6_	182.079	(M − H)−	181.0717	181.0717	0.27	179.0563, 133.0142
**3**	0.648	Gulonic acid (M)	C_6_H_12_O_7_	196.0582	(M − H)−	195.0508	195.0508	0.34	191.0562, 179.0563, 165.0404, 133.0142
**4**	0.683	Shikimic acid (H, M)	C_7_H_10_O_5_	174.0532	(M − H)−	173.0458	173.0458	−0.36	191.0562, 133.0142
**5**	0.687	D-malic acid (H, M)	C_4_H_6_O_5_	134.0215	(M − H)−	133.0142	133.0142	0.13	112.9856
**6**	0.992	Succinic acid (H)	C_4_H_6_O_4_	118.0264	(M − H)−	117.0191	117.0191	2.06	112.9856
**7**	1.961	Pantothenic acid (H, M)	C_9_H_17_NO_5_	219.1108	(M + H)+	220.1183	220.1181	−0.80	205.0863, 194.1177, 186.9566, 158.0030, 121.0509
**8**	2.402	Damascenone (M)	C_13_H_18_O	190.1357	(M + H)+	191.1430	191.1430	0.20	158.0030, 141.9587, 121.0509
**9**	2.792	Chlorogenic acid (H, M)	C_16_H_18_O_9_	354.0951	(M + H)+	355.1022	355.1023	0.06	311.1854, 279.1593, 231.2068, 194.1177, 158.0031, 121.0509
**10**	3.430	3-Hydoxybenzoic acid (H, M)	C_7_H_6_O_3_	138.0318	(M − H)−	137.0245	137.0246	−0.79	112.9856
**11**	4.503	Chlorogenoquinone (M)	C_16_H_16_O_9_	352.0793	(M − H)−	351.0719	351.0720	0.35	191.0560
**12**	4.727	Esculetin (H, M)	C_9_H_6_O_4_	178.0267	(M − H)−	177.0194	177.0194	−0.69	147.0298
**13**	5.490	Feruloylagmatine (M)	C_15_H_22_N_4_O_3_	306.1693	(M + H)+	307.1766	307.1765	−0.46	262.1801, 231.2067, 194.1178, 158.0030, 121.0509
**14**	5.756	Kaempferol 3-(2G-xylosylrutinoside)-7-glucoside (M)	C_38_H_48_O_24_	888.2530	(M − H)−	887.2452	887.2455	0.59	435.2235
**15**	7.022	Isopentyl gentiobioside (M)	C_17_H_32_O_11_	412.1938	(M + HCOO)¯	457.1920	457.1919	1.6	427.1821
**16**	7.086	Kaempferol 3-*O*-rutinoside (2) or nicotiflorin (M, S, isolated)	C_27_H_30_O_15_	594.1580	(M + H)+	595.1641	595.1650	0.82	536.1648, 470.3695, 372.2498, 307.1764, 279.1595, 231.2067, 194.1177, 158.0030, 121.0509
**17**	8.867	Quercetin 3-O-(β-D-apiofuranosyl(1→2)-α-rhamnopyranosyl(1→6)-β-D-glucopyranoside) (5) (M, isolated)	C_32_H_38_O_20_	742.1952	(M − H)−	741.1880	741.1880	0.65	No fragment ions observed
**18**	9.387	Rutin (1) (H, M, S, isolated)	C_27_H_30_O_16_	610.1524	(M + H)+	611.1578	611.1578	1.6	536.1649, 373.2333, 311.1854, 279.1596, 231.2066, 194.1177, 158.0030, 121.0509
**19**	9.388	Herbacetin (M)	C_15_H_10_O_7_	302.0426	(M + H)+	303.0501	303.0499	0.32	279.1596, 231.2066, 194.1177, 158.0030, 121.0509
**20**	9.660	Kaempferol 3-O-(β-D-apiofuranosyl(1→2)-α-rhamnopyranosyl(1→6)-β-D-galactopyranoside (7) (M, isolated)	C_32_H_38_O_19_	726.1998	(M − H)−	725.1926	725.1926	1.22	609.1462
**21**	9.696	Quercetin 3-*O*-glucoside (M)	C_21_H_20_O_12_	464.0957	(M + H)+	465.1028	465.1028	−0.57	453.3431, 393.2476, 357.2384, 279.1595, 231.2067, 194.1177, 158.0030, 121.0509
**22**	10.444	Luteolin (M)	C_15_H_10_O_6_	286.0478	(M + H)+	287.0550	287.0552	−0.13	279.1595, 262.1801, 231.2067, 194.1178, 158.0030, 121.0509
**23**	10.800	Citrusin B (M)	C_27_H_36_O_13_	568.2157	(M − H)−	567.2087	567.2090	−0.26	146.9657
**24**	11.171	Astragalin (M)	C_21_H_20_O_11_	448.1005	(M + H)+	449.1080	449.1079	0.04	393.2466, 317.0603, 279.1594, 231.2066, 194.1177, 158.0031, 121.0509
**25**	16.837	Jasmonic acid (H, M)	C_12_H_18_O_3_	210.1260	(M + H)+	211.1324	211.1324	−2.10	194.1177, 180.1361, 158.1540, 121.0509
**26**	17.190	2-Phenylpropionic acid (M)	C_9_H_10_O_2_	150.0680	(M + H)+	151.0753	151.0753	0.53	130.1593, 124.0872, 121.0509, 118.0863
**27**	17.199	Rishitin (M)	C_14_H_22_O_2_	222.1619	(M − H)−	221.1546	221.1546	0.52	132.9234
**28**	17.683	3,8-Dihydroxy-6-methoxy-7(11)-eremophilen-12,8-olide (M)	C_16_H_24_O_5_	296.1618	(M − H)¯	295.1544	295.1547	1.97	194.0824, 132.9234
**29**	17.849	Gingerglycolipid A (M)	C_33_H_56_O_14_	676.3665	(M + HCOO)−	721.3646	721.3646	0.77	339.1997, 311.1687, 132.9234
**30**	18.296	Gingerglycolipid B (M)	C_33_H_58_O_14_	678.3828	(M + HCOO)−	723.3810	723.3810	−0.19	311.1687, 132.9234
**31**	18.563	Isocurcumenol (M)	C_15_H_22_O_2_	234.1619	(M − H)−	233.1546	233.1546	0.52	132.9235
**32**	18.635	Isokobusone (H, M)	C_14_H_22_O_2_	222.1620	(M − H)−	221.1546	221.1546	0.09	132.9235

**Table 2:** α-Glucosidase inhibitory activity of glycosides **1**–**7**. Results are expressed as mean ± s.d. of a triplicate experiment.

Compound	α-Glucosidase Inhibitory Activity, IC_50_ (µM)
**1**	243.4 ± 5.29
**2**	235.8 ± 27.67
**3**	195.4 ± 18.13
**4** (and **1**), the ratio of 1.00:0.79	342.2 ± 28.72
**5**	431.0 ± 17.64
**6**	455.6 ± 26.00
**7**	432.5 ± 18.88
Acarbose	2023.3 ± 17.34

**Table 3:** Virucidal activity of glycosides **1**–**6**. Results are expressed as mean ± s.d. of a quadruplicate experiment.

Compound	Concentration of Compound (µg/mL)	Cytotoxicity (% Cell Viability)	Virucidal Activity Log Reduction
**1**	125.0	77.2 ± 1.28	1.80 ± 0.105
	62.5	83.8 ± 2.02	1.52 ± 0.045
	31.3	86.5 ± 2.16	1.34 ± 0.030
	15.6	94.7 ± 3.93	1.34 ± 0.044
	7.8	97.8 ± 0.31	1.32 ± 0.032
	3.9	99.3 ± 3.45	1.20 ± 0.025
**2**	125.0	78.0 ± 1.75	1.95 ± 0.124
	62.5	86.5 ± 3.82	1.82 ± 0.091
	31.3	88.5 ± 2.67	1.68 ± 0.046
	15.6	92.3 ± 1.95	1.66 ± 0.071
	7.8	96.3 ± 5.13	1.65 ± 0.084
	3.9	98.1 ± 1.34	1.66 ± 0.071
**3**	125.0	73.3 ± 0.90	1.98 ± 0.088
	62.5	83.6 ± 0.74	1.91 ± 0.062
	31.3	88.7 ± 0.21	1.95 ± 0.124
	15.6	89.7 ± 0.82	1.91 ± 0.062
	7.8	92.8 ± 4.84	1.66 ± 0.071
	3.9	95.6 ± 6.25	1.63 ± 0.069
A mixture of **4** and **1** with the ratio of 1.00:0.79	500.0	83.4 ± 1.63	2.41 ± 0.000
	250.0	84.6 ± 2.08	2.01 ± 0.102
	125.0	89.9 ± 1.88	1.90 ± 0.142
	62.5	93.8 ± 1.83	1.84 ± 0.108
	31.3	94.7 ± 0.97	1.81 ± 0.091
	15.6	96.9 ± 0.54	1.87 ± 0.072
**5**	500.0	82.9 ± 2.06	2.11 ± 0.198
	250.0	83.7 ± 1.20	2.01 ± 0.239
	125.0	85.8 ± 2.87	1.97 ± 0.088
	62.5	90.0 ± 3.08	1.90 ± 0.062
	31.3	94.1 ± 1.85	1.84 ± 0.108
	15.6	95.8 ± 4.22	1.87 ± 0.072
**6**	500.0	86.4 ± 2.06	1.97 ± 0.042
	250.0	88.6 ± 0.43	1.93 ± 0.124
	125.0	89.5 ± 0.83	1.93 ± 0.124
	62.5	93.7 ± 4.87	1.93 ± 0.124
	31.3	95.7 ± 1.63	1.93 ± 0.124
	15.6	97.0 ± 2.99	1.93 ± 0.124

## Supplementary Materials

The following are available online at https://www.mdpi.com/article/10.3390/foods10123041/s1, Figure S1: ^1^H NMR (in methanol-*d4*) of methanolic crude extract of *C**. grandis* leaves (after removing fat by hexane extraction); Figure S2: TIC chromatogram of methanolic crude extract of *C**. grandis* in positive ion mode; Figure S3: Molecular networking of methanolic crude extract of *C**. grandis* in positive ion mode; Figure S4: ^1^H NMR (400 MHz) spectrum of rutin (**1**) in methanol-*d4*; Figure S5: ^13^C NMR (100 MHz) spectrum of rutin (**1**) in methanol-*d4*; Figure S6: HRESI-MS spectrum of rutin (**1**) in a positive ionization mode; Figure S7: ^1^H NMR (400 MHz) spectrum of kaempferol 3-*O*-rutinoside (**2**) in methanol-*d4*; Figure S8: ^13^C NMR (100 MHz) spectrum of kaempferol 3-*O*-rutinoside (**2**) in methanol-*d4*; Figure S9: HRESI-MS spectrum of kaempferol 3-*O*-rutinoside (**2**) in a positive ionization mode; Figure S10: ^1^H NMR (400 MHz) spectrum of kaempferol 3-*O*-robinobioside (**3**) in methanol-*d4;* Figure S11: ^13^C NMR (100 MHz) spectrum of kaempferol 3-*O*-robinobioside (**3**) in methanol-*d4*; Figure S12: HRESI-MS spectrum of kaempferol 3-*O*-robinobioside (**3**) in a positive ionization mode; Figure S13: 1H NMR (400 MHz) spectrum of a mixture of quercetin 3-*O*-robinobioside (**4**) and rutin (**1**) in methanol-*d4*; Figure S14: 13C NMR (100 MHz) spectrum of a mixture of quercetin 3-*O*-robinobioside (**4**) and rutin (**1**) in methanol-*d4*; Figure S15: HRESI-MS spectrum of a mixture of quercetin 3-*O*-robinobioside (**4**) and rutin (**1**) in a positive ionization mode; Figure S16: ^1^H NMR (400 MHz) spectrum of quercetin 3-*O*-β-D-apiofuranosyl-(1→2)-[α-L-rhamnopyranosyl-(1→6)]-β-D-glucopyranoside (**5**) in methanol-*d4*; Figure S17: ^13^C NMR (100 MHz) spectrum of quercetin 3-*O*-β-D-apiofuranosyl-(1→2)-[α-L-rhamnopyranosyl-(1→6)]-β-D-glucopyranoside (**5**) in methanol-*d4*; Figure S18: HRESI-MS spectrum of quercetin 3-*O*-β-D-apiofuranosyl-(1→2)-[α-L-rhamnopyranosyl-(1→6)]-β-D-glucopyranoside (**5**) in a positive ionization mode; Figure S19: ^1^H NMR (400 MHz) spectrum of kaempferol 3-*O*-β-D-apiofuranosyl-(1→2)-[α-L-rhamnopyranosyl-(1→6)]-β-D-glucopyranoside (**6**) in methanol-*d4*; Figure S20: ^13^C NMR (100 MHz) spectrum of kaempferol 3-*O*-β-D-apiofuranosyl-(1→2)-[α-L-rhamnopyranosyl-(1→6)]-β-D-glucopyranoside (**6**) in methanol-*d4*; Figure S21: HRESI-MS spectrum of kaempferol 3-*O*-β-D-apiofuranosyl-(1→2)-[α-L-rhamnopyranosyl-(1→6)]-β-D-glucopyranoside (**6**) in a positive ionization mode; Figure S22: ^1^H NMR (400 MHz) spectrum of kaempferol 3-*O*-β-D-apiofuranosyl-(1→2)-[α-L-rhamnopyranosyl-(1→6)]-β-D-galactopyranoside (**7**) in methanol-*d4*; Figure S23: ^13^C NMR (100 MHz) spectrum of kaempferol 3-*O*-β-D-apiofuranosyl-(1→2)-[α-L-rhamnopyranosyl-(1→6)]-β-D-galactopyranoside (**7**) in methanol-*d4*; Figure S24: HSQC spectrum of kaempferol 3-*O*-β-D-apiofuranosyl-(1→2)-[α-L-rhamnopyranosyl-(1→6)]-β-D-galactopyranoside (**7**) in methanol-*d4*; Figure S25: HMBC spectrum of kaempferol 3-*O*-β-D-apiofuranosyl-(1→2)-[α-L-rhamnopyranosyl-(1→6)]-β-D-galactopyranoside (**7**) in methanol-*d4*; Figure S26: HRESI-MS spectrum of kaempferol-3-*O*-β-D-apiofuranosyl-(1→2)-[α-L-rhamnopyranosyl-(1→6)]-β-D-galactopyranoside (**7**) in a positive ionization mode; Figure S27: Dose-dependent inhibition of *C*. *grandis* crude extract towards α-glucosidase enzyme; Table S1: ^1^H (400 MHz) and ^13^C NMR (100 MHz) data of compounds **1**–**4** in methanol-*d*4; Table S2: ^1^H (400 MHz) and ^13^C NMR (100 MHz) data of compounds **5**–**7** in methanol-*d4.*
