# Supplementary material for: LC-QTOF-MS/MS Based Molecular Networking Approach for the Isolation of α-Glucosidase Inhibitors and Virucidal Agents from Coccinia grandis (L.) Voigt
Source: Foods. 2021 Dec 7;10(12):3041. doi: 10.3390/foods10123041 (PMC8701318; doi:10.3390/foods10123041)
Supplement: Supplementary file 1 [file foods-10-03041-s001.zip › foods-1481668-SI.pdf]

Supplementary materials

LC-QTOF-MS/MS based molecular networking approach for the isolation of α-glucosidase inhibitors and virucidal agents from *Coccinia grandis* (L.) Voigt

Maharani A. Astiti, Akanitt Jittmittraphap, Pornsawan Leaungwutiwong, Nopporn Chutiwitoonchai, Patcharee Pripdeevech, Chulabhorn Mahidol, Somsak Ruchirawat, Prasat Kittakoop

CONTENTS

Figure S1. ^{1}H NMR (in methanol-*d4*) of a methanolic crude extract of *C. grandis* leaves (after removing fat by hexane extraction)
Figure S2. TIC chromatogram of a methanolic crude extract of *C. grandis* in a positive ion mode
Figure S3. Molecular networking of methanolic crude extract of *C. grandis* in a positive ion mode
Figure S4. ^{1}H NMR (400 MHz) spectrum of rutin (**1**) in methanol-*d4*
Figure S5. ^{13}C NMR (100 MHz) spectrum of rutin (**1**) in methanol-*d4*
Figure S6. HRESI-MS spectrum of rutin (**1**) in a positive ionization mode
Figure S7. ^{1}H NMR (400 MHz) spectrum of kaempferol 3-*O*-rutinoside (**2**) in methanol-*d4*
Figure S8. ^{13}C NMR (100 MHz) spectrum of kaempferol 3-*O*-rutinoside (**2**) in methanol-*d4*
Figure S9. HRESI-MS spectrum of kaempferol 3-*O*-rutinoside (**2**) in a positive ionization mode
Figure S10. ^{1}H NMR (400 MHz) spectrum of kaempferol 3-*O*-robinobioside (**3**) in methanol-*d4*
Figure S11. ^{13}C NMR (100 MHz) spectrum of kaempferol 3-*O*-robinobioside (**3**) in methanol-*d4*
Figure S12. HRESI-MS spectrum of kaempferol 3-*O*-robinobioside (**3**) in a positive ionization mode
Figure S13. ^{1}H NMR (400 MHz) spectrum of a mixture of quercetin 3-*O*-robinobioside (**4**) and rutin (**1**) in methanol-*d4*
Figure S14. ^{13}C NMR (100 MHz) spectrum of a mixture of quercetin 3-*O*-robinobioside (**4**) and rutin (**1**) in methanol-*d4*
Figure S15. HRESI-MS spectrum of a mixture of quercetin 3-*O*-robinobioside (**4**) and rutin (**1**) in a positive ionization mode
Figure S16. ^{1}H NMR (400 MHz) spectrum of quercetin 3-*O*-β-D-apiofuranosyl-(1→2)-[α-L-rhamnopyranosyl-(1→6)]-β-D-glucopyranoside (**5**) in methanol-*d4*
Figure S17. ^{13}C NMR (100 MHz) spectrum of quercetin 3-O-β-D-apiofuranosyl-(1→2)-[α-L-rhamnopyranosyl-(1→6)]-β-D-glucopyranoside (**5**) in methanol-*d4*
Figure S18. HRESI-MS spectrum of quercetin 3-*O*-β-D-apiofuranosyl-(1→2)-[α-L-rhamnopyranosyl-(1→6)]-β-D-glucopyranoside (**5**) in a positive ionization mode
Figure S19. ^{1}H NMR (400 MHz) spectrum of kaempferol 3-*O*-β-D-apiofuranosyl-(1→2)-[α-L-rhamnopyranosyl-(1→6)]-β-D-glucopyranoside (**6**) in methanol-*d4*
Figure S20. ^{13}C NMR (100 MHz) spectrum of kaempferol 3-*O*-β-D-apiofuranosyl-(1→2)-[α-L-rhamnopyranosyl-(1→6)]-β-D-glucopyranoside (**6**) in methanol-*d4*
Figure S21. HRESI-MS spectrum of kaempferol 3-*O*-β-D-apiofuranosyl-(1→2)-[α-L-rhamnopyranosyl-(1→6)]-β-D-glucopyranoside (**6**) in a positive ionization mode
Figure S22. ^{1}H NMR (400 MHz) spectrum of kaempferol 3-*O*-β-D-apiofuranosyl-(1→2)-[α-L-rhamnopyranosyl-(1→6)]-β-D-galactopyranoside (**7**) in methanol-*d4*
Figure S23. ^{13}C NMR (100 MHz) spectrum of kaempferol 3-*O*-β-D-apiofuranosyl-(1→2)-[α-L-rhamnopyranosyl-(1→6)]-β-D-galactopyranoside (**7**) in methanol-*d4*

Figure S24. HSQC spectrum of kaempferol 3-*O*-β-D-apiofuranosyl-(1→2)-[α-L-rhamnopyranosyl-(1→6)]-β-D-galactopyranoside (**7**) in methanol-*d4*

Figure S25. HMBC spectrum of kaempferol 3-*O*-β-D-apiofuranosyl-(1→2)-[α-L-rhamnopyranosyl-(1→6)]-β-D-galactopyranoside (**7**) in methanol-*d4*

Figure S26. HRESI-MS spectrum of kaempferol-3-*O*-β-D-apiofuranosyl-(1→2)-[α-L-rhamnopyranosyl-(1→6)]-β-D-galactopyranoside (**7**) in a positive ionization mode

Figure S27. Dose-dependent inhibition of *C. grandis* crude extract towards α-glucosidase enzyme

Table S1. ^{1}H (400 MHz) and ^{13}C NMR (100 MHz) data of compounds **1-4** in methanol-*d4*

Table S2. ^{1}H (400 MHz) and ^{13}C NMR (100 MHz) data of compounds **5-7** in methanol-*d4*

Figure S1. ¹H NMR (in methanol-*d4*) of a methanolic crude extract of *C. grandis* leaves (after removing fat by hexane extraction)

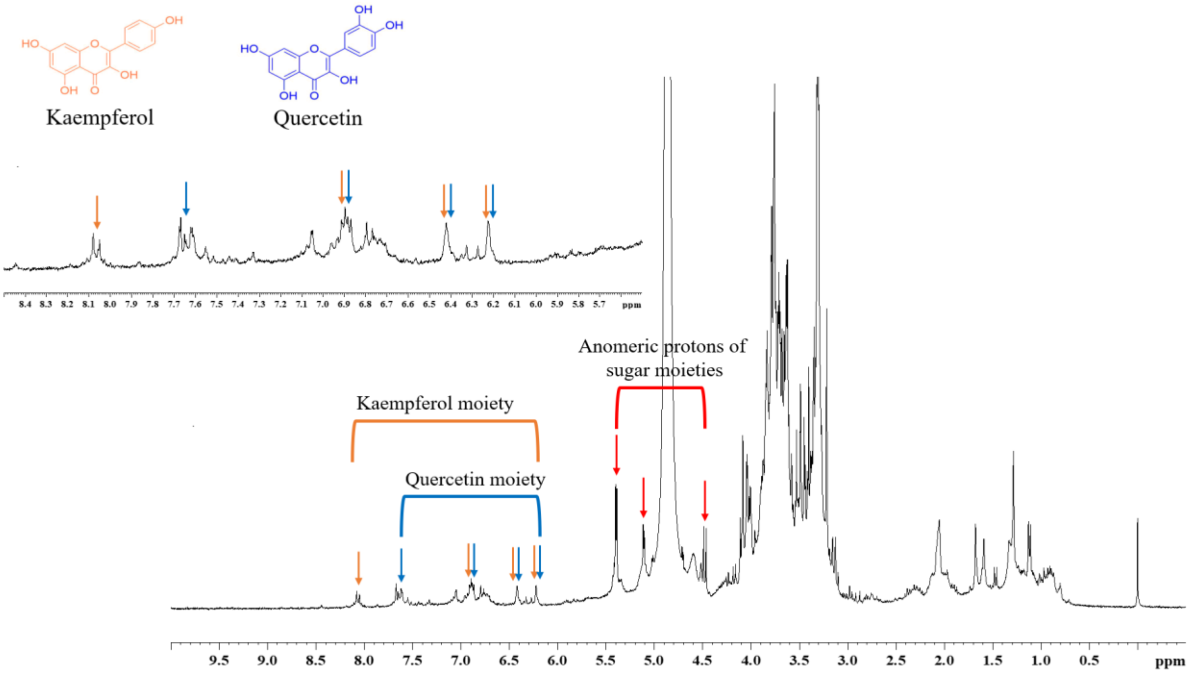

Figure S2. TIC chromatogram of a methanolic crude extract of *C. grandis* in a positive ion mode

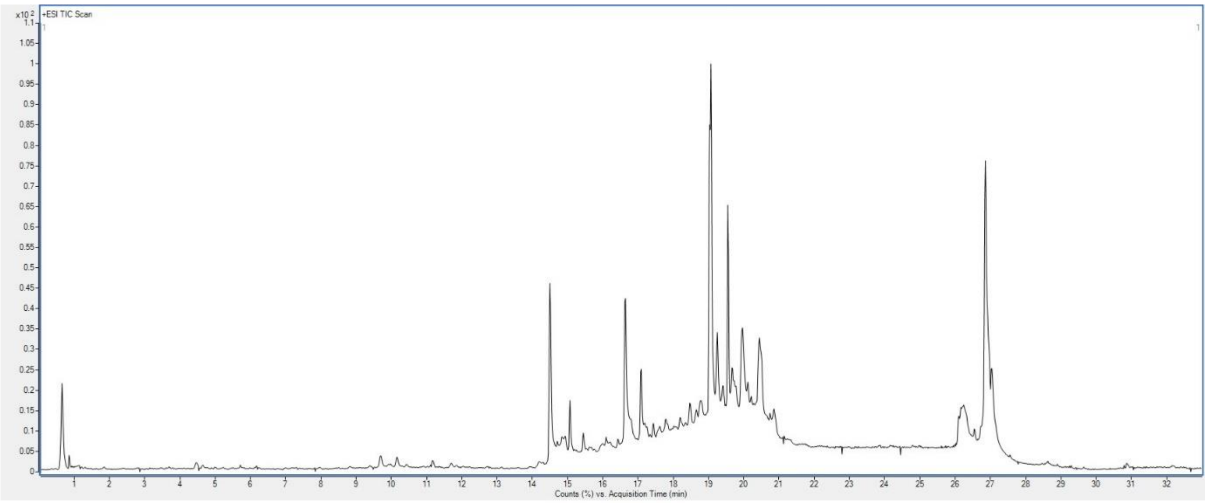

Figure S3. Molecular networking of methanolic crude extract of *C. grandis* in a positive ion mode

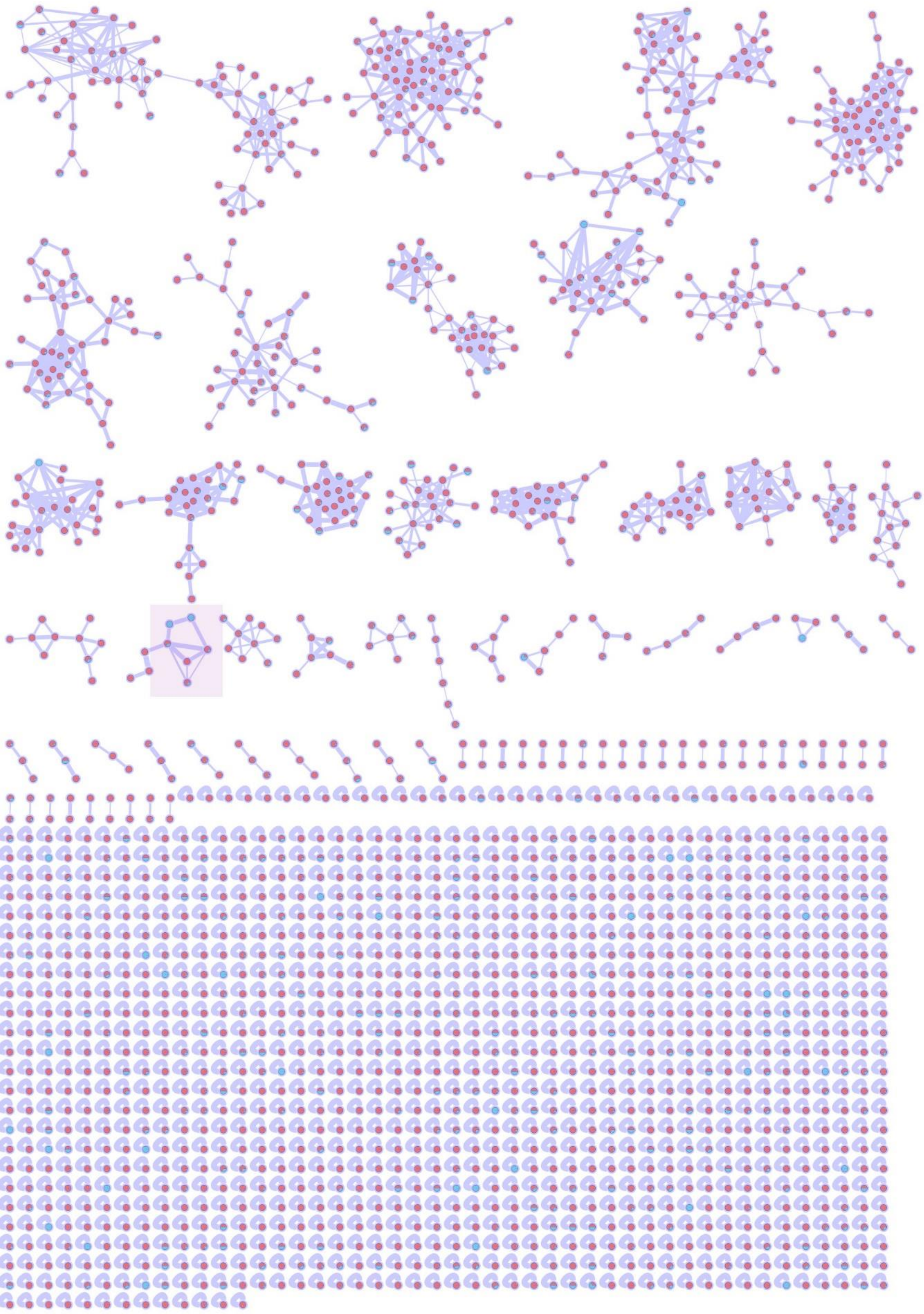

Figure S4. ^{1}H NMR (400 MHz) spectrum of rutin (**1**) in methanol-*d4*

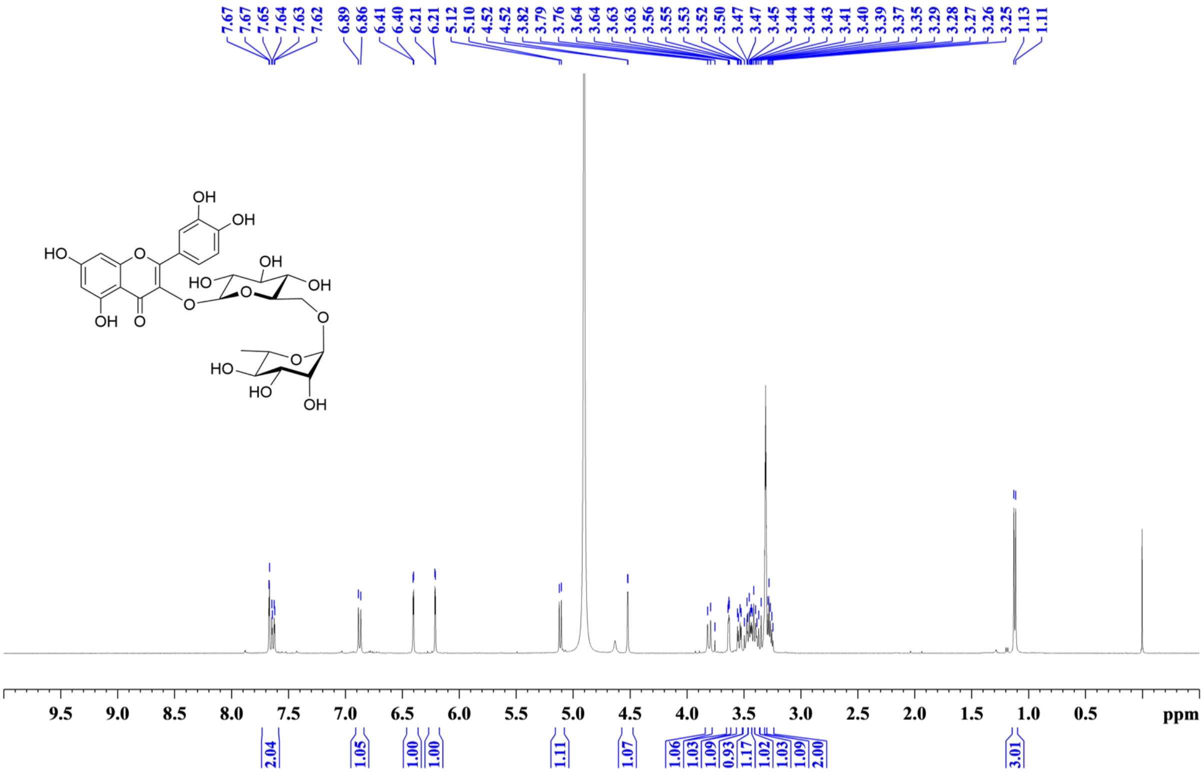

Figure S5. ^{13}C NMR (100 MHz) spectrum of rutin (**1**) in methanol-*d4*

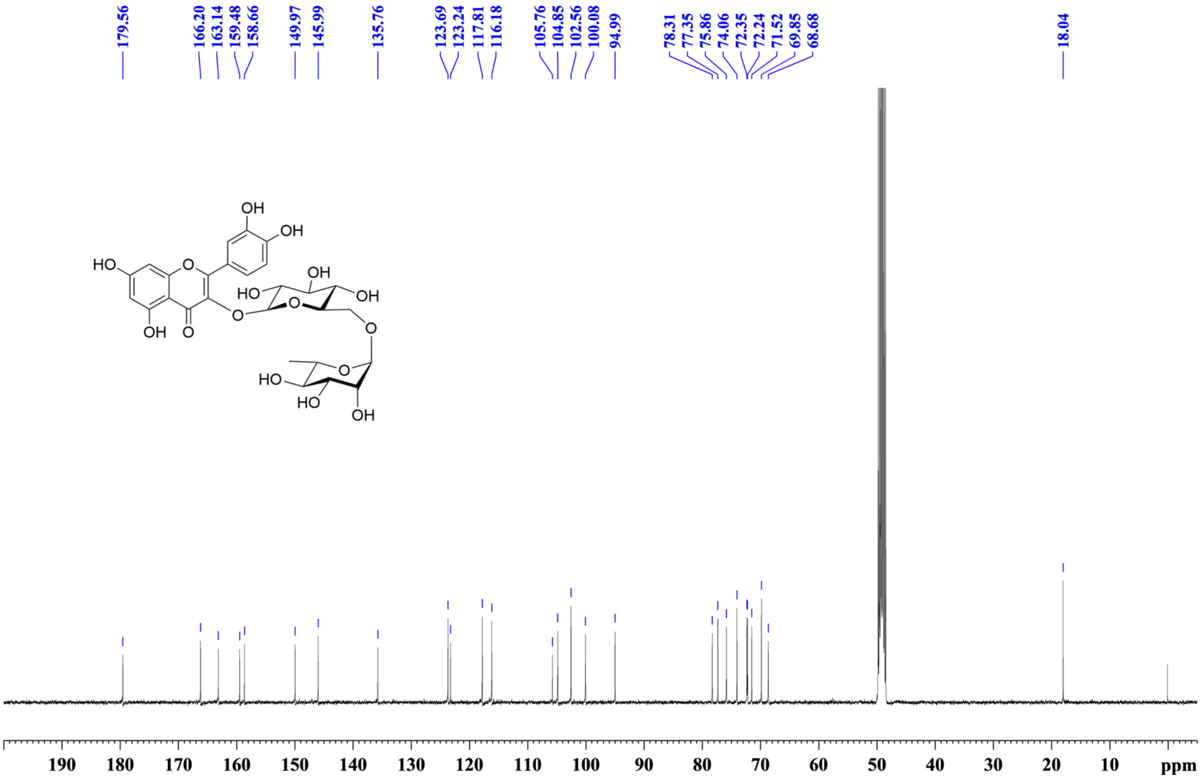

Figure S6. HRESI-MS spectrum of rutin (**1**) in a positive ionization mode

Rutin (**1**) had the observed ion at *m/z* 633.1424 [M+Na]$^+$, calcd for [$C_{27}H_{30}O_{16}{}^{23}Na$]$^+$, 633.1426, $\Delta_{m/z}$ = -0.33 ppm, with molecular formula of $C_{27}H_{30}O_{16}$.

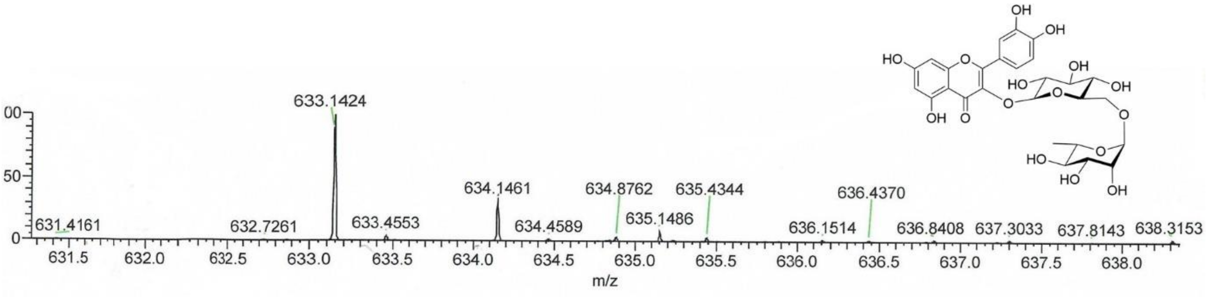

Figure S7. ^{1}H NMR (400 MHz) spectrum of kaempferol 3-*O*-rutinoside (**2**) in methanol-*d4*

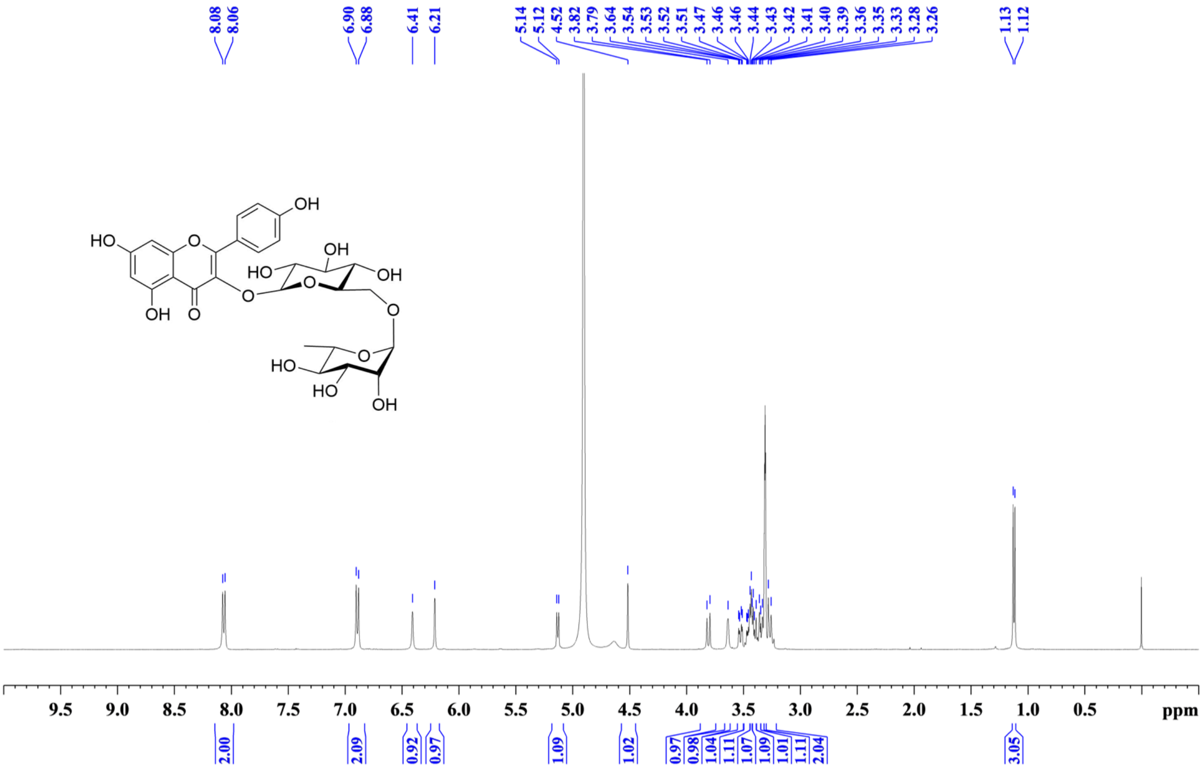

Figure S8. ^{13}C NMR (100 MHz) spectrum of kaempferol 3-*O*-rutinoside (**2**) in methanol-*d4*

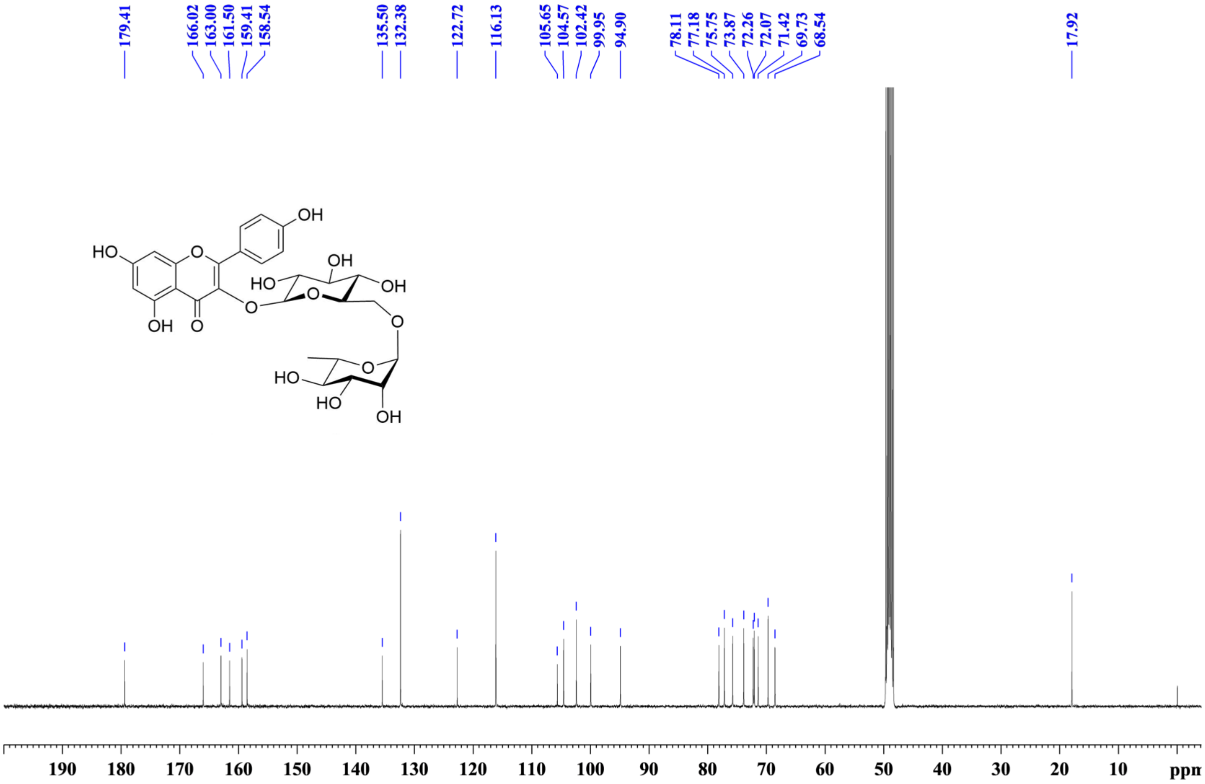

Figure S9. HRESI-MS spectrum of kaempferol 3-*O*-rutinoside (**2**) in a positive ionization mode

Kaempferol 3-*O*-rutinoside (**2**) had the observed ion at *m/z* 617.1471 [M+Na]$^+$, calcd for [$C_{27}H_{30}O_{15}{}^{23}Na$]$^+$, 617.1477, $\Delta_{m/z}$ = -0.97 ppm, with molecular formula of $C_{27}H_{30}O_{15}$.

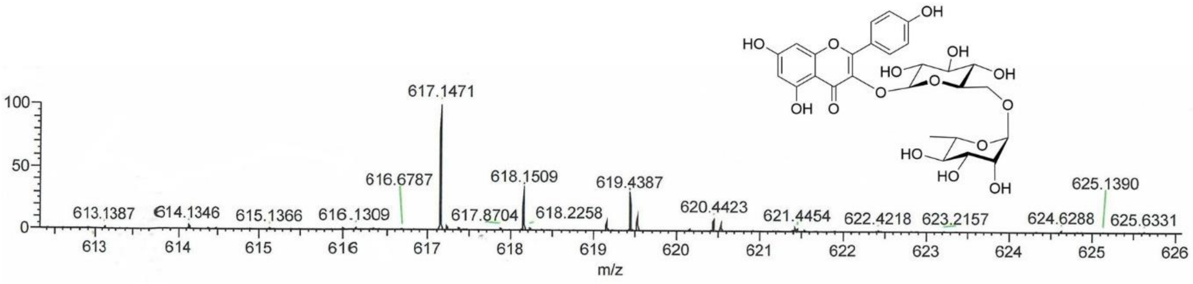

Figure S10. ^{1}H NMR (400 MHz) spectrum of kaempferol 3-*O*-robinobioside (**3**) in methanol-*d4*

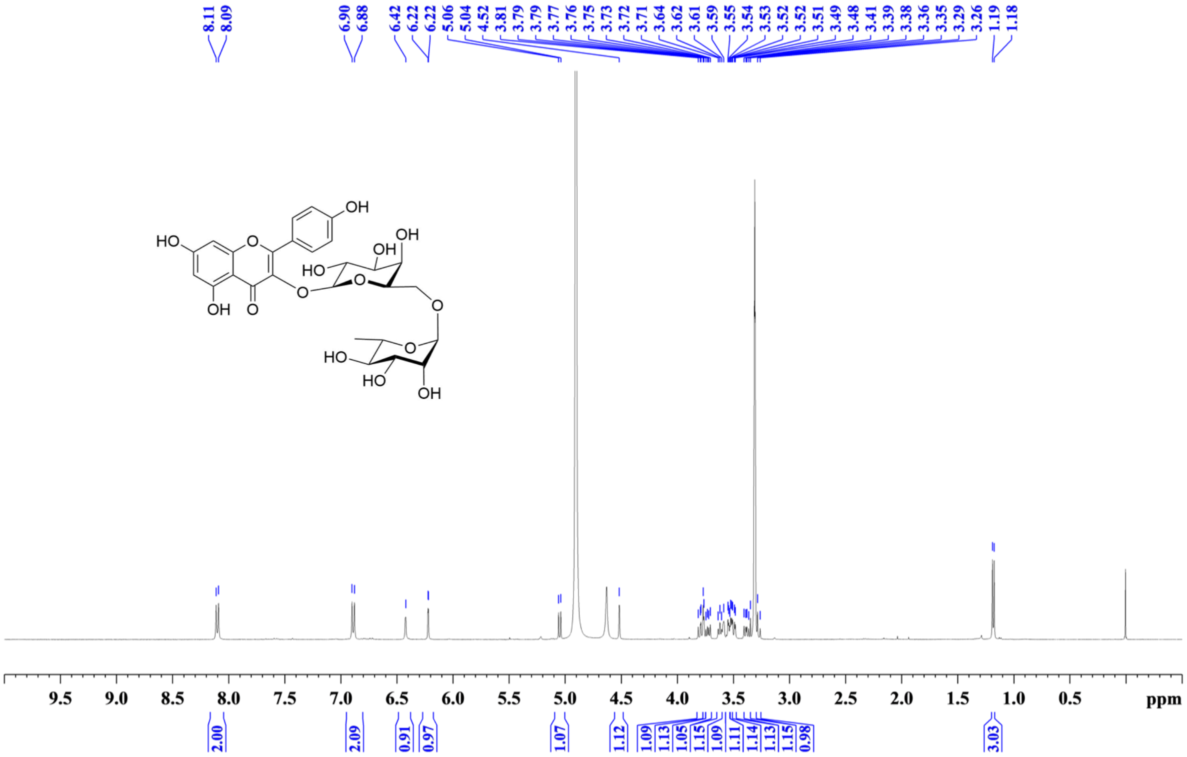

Figure S11. ^{13}C NMR (100 MHz) spectrum of kaempferol 3-*O*-robinobioside (**3**) in methanol-*d4*

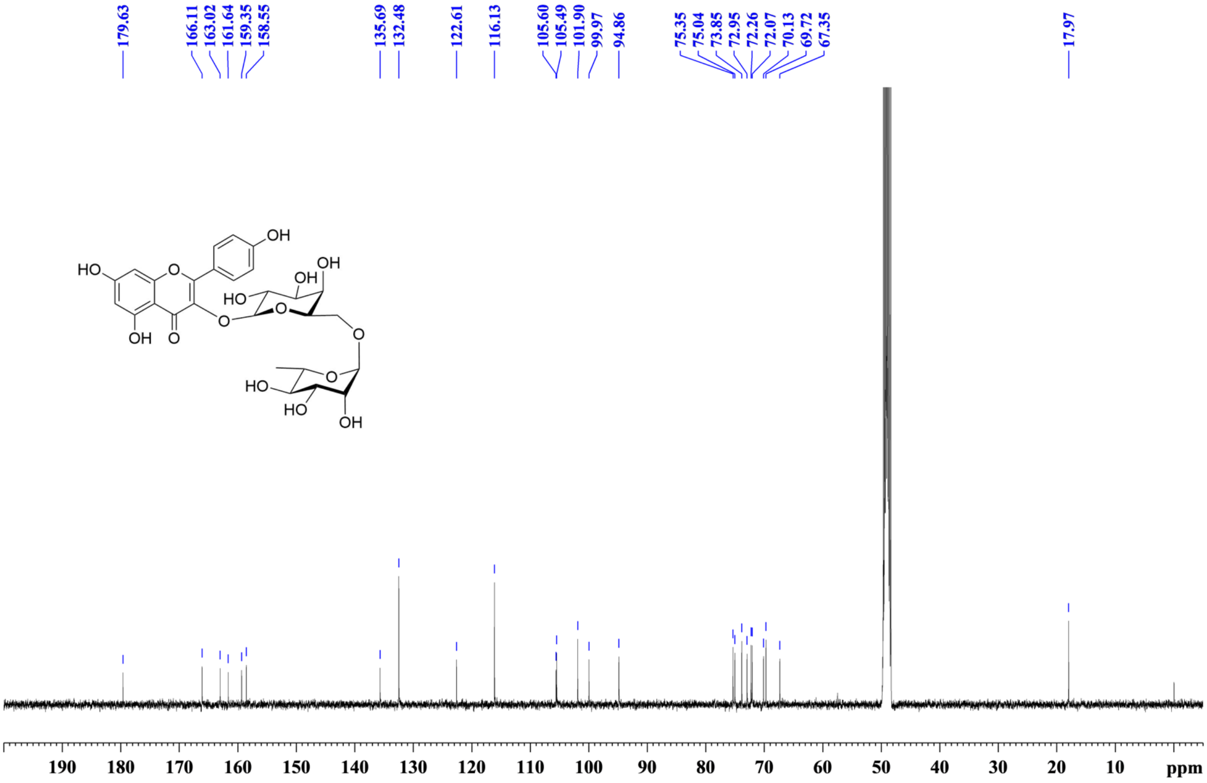

Figure S12. HRESI-MS spectrum of kaempferol 3-*O*-robinobioside (**3**) in a positive ionization mode

Kaempferol 3-*O*-robinobioside (**3**) had the observed ion at *m/z* 617.1486 $[M+Na]^+$, calcd for $[C_{27}H_{30}O_{15}{}^{23}Na]^+$, 617.1477, $\Delta_{m/z}$ = 1.51 ppm, with molecular formula of $C_{27}H_{30}O_{15}$.

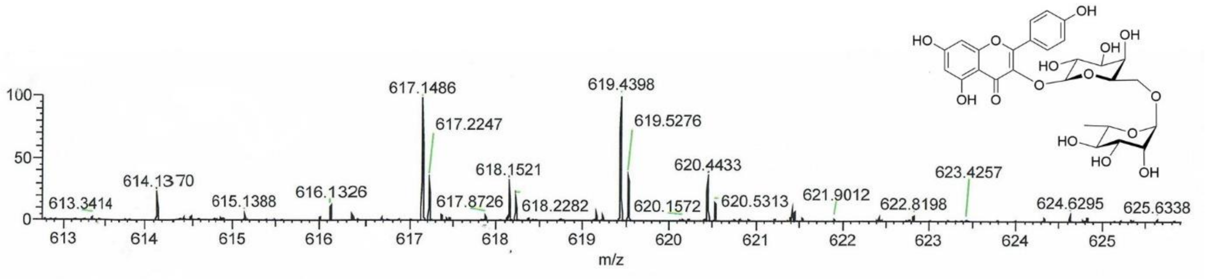

Figure S13. ^{1}H NMR (400 MHz) spectrum of a mixture of quercetin 3-*O*-robinobioside (**4**) and rutin (**1**) in methanol-*d4*

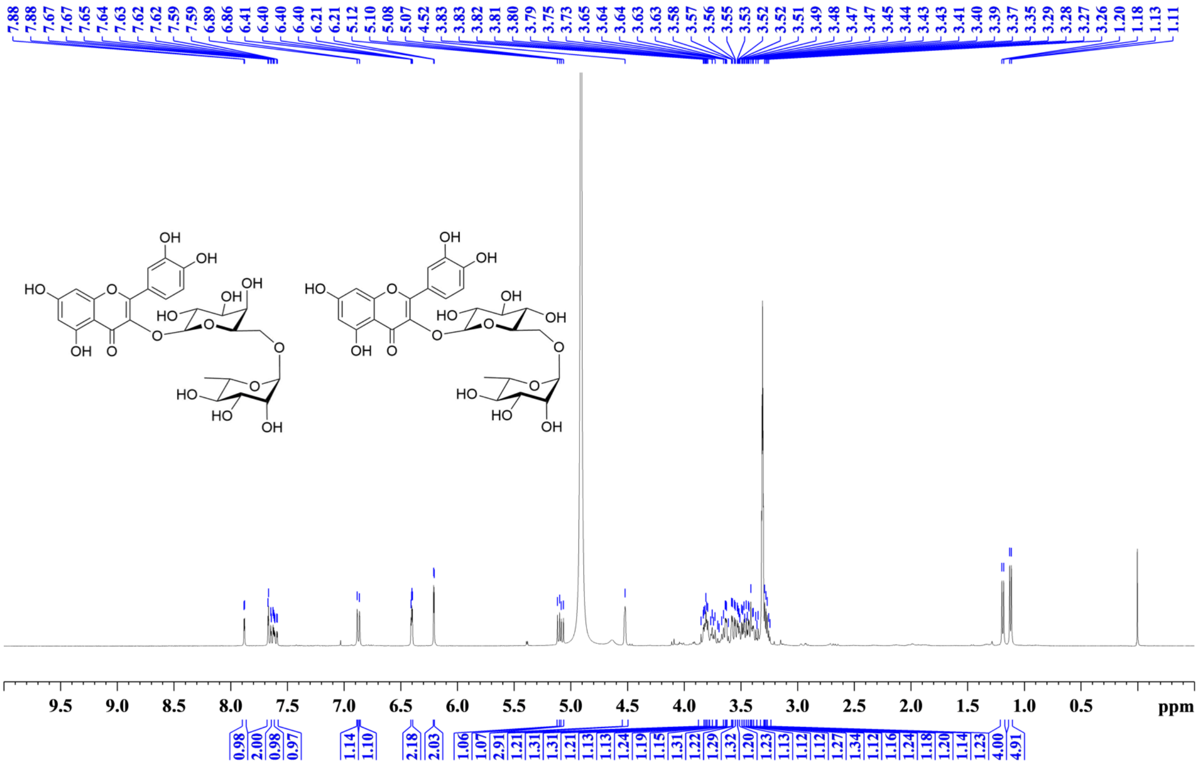

Figure S14. ^{13}C NMR (100 MHz) spectrum of a mixture of quercetin 3-*O*-robinobioside (**4**) and rutin (**1**) in methanol-*d4*

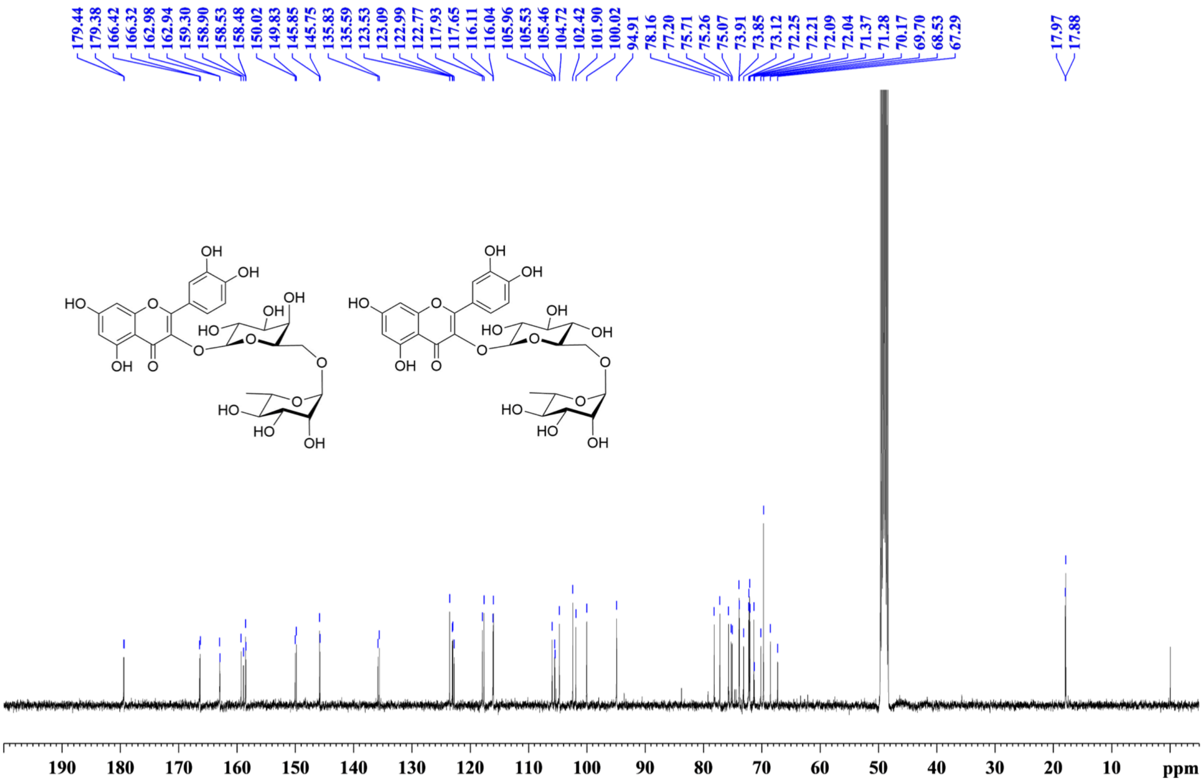

Figure S15. HRESI-MS spectrum of a mixture of quercetin 3-*O*-robinobioside (**4**) and rutin (**1**) in a positive ionization mode

A mixture of quercetin 3-*O*-robinobioside (**4**) and rutin (**1**) had the observed ion at *m/z* 633.1434 [M+Na]$^+$, calcd for [$C_{27}H_{30}O_{16}{}^{23}Na$]$^+$, 633.1426, $\Delta_{m/z}$ = 1.21 ppm, with molecular formula of $C_{27}H_{30}O_{16}$.

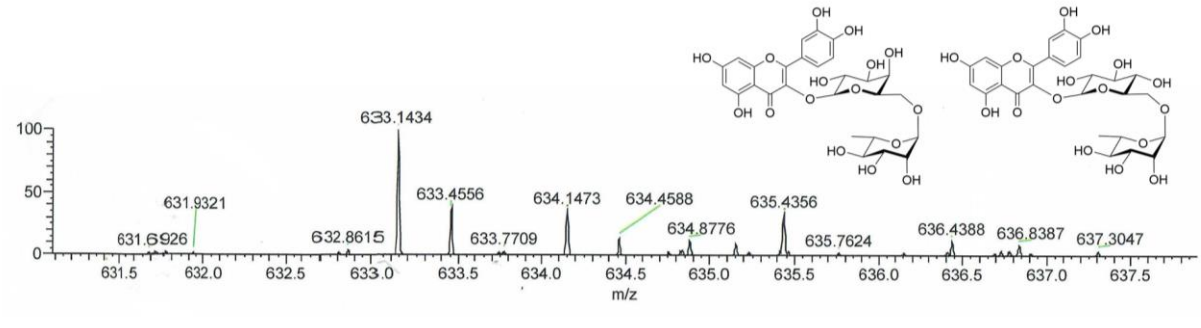

Figure S16. ^{1}H NMR (400 MHz) spectrum of quercetin 3-*O*-β-D-apiofuranosyl-(1→2)-[α-L-rhamnopyranosyl-(1→6)]-β-D-glucopyranoside (**5**) in methanol-*d4*

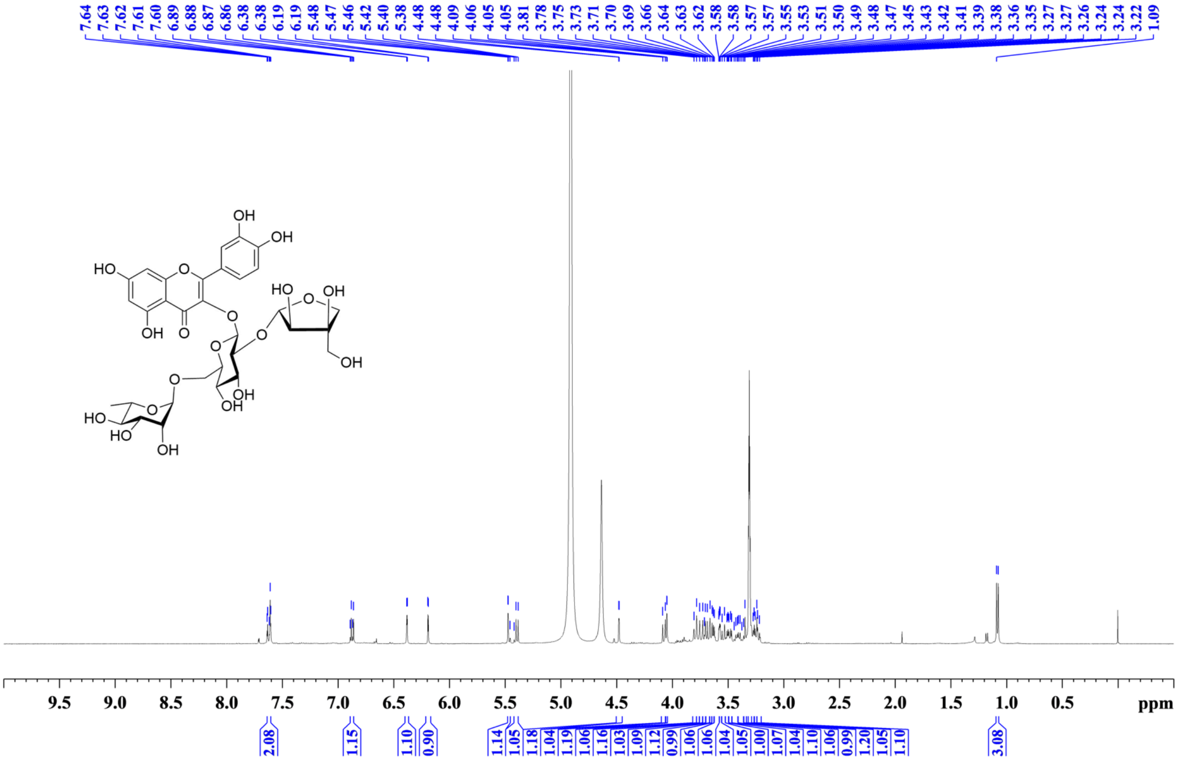

Figure S17. ^{13}C NMR (100 MHz) spectrum of quercetin 3-*O*-β-D-apiofuranosyl-(1→2)-[α-L-rhamnopyranosyl-(1→6)]-β-D-glucopyranoside (**5**) in methanol-*d4*

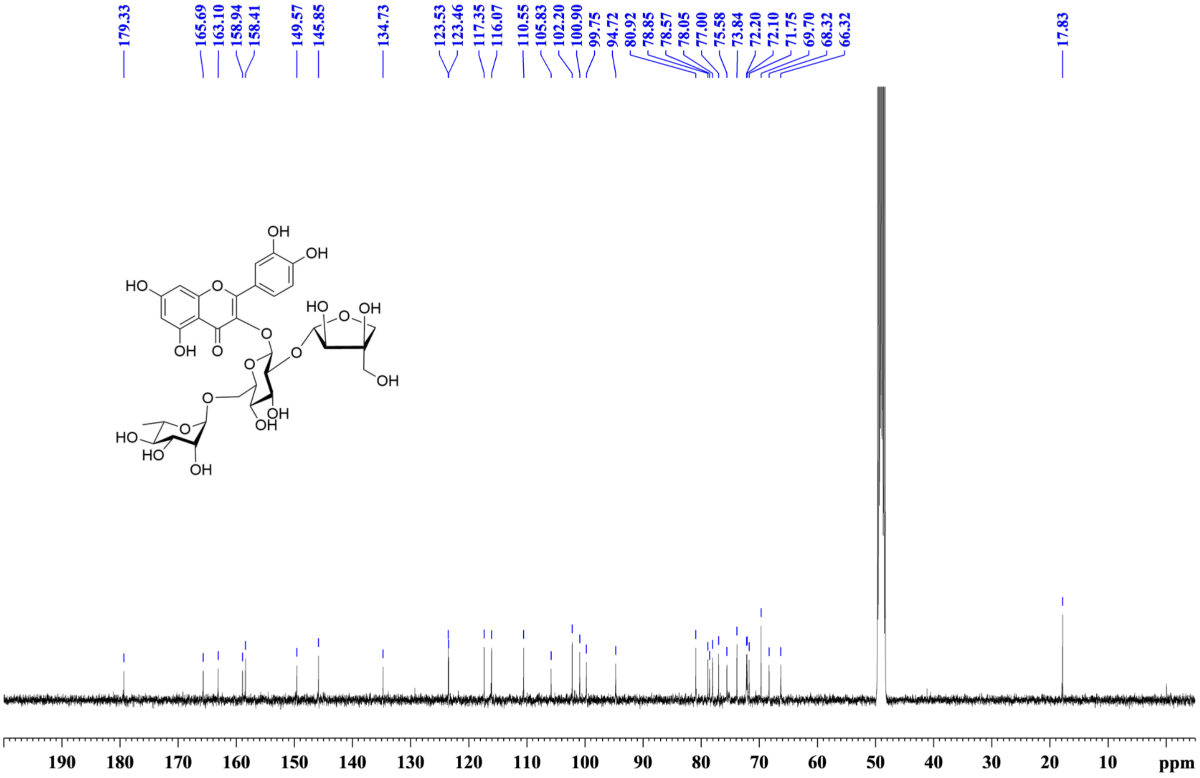

Figure S18. HRESI-MS spectrum of quercetin 3-*O*-β-D-apiofuranosyl-(1→2)-[α-L-rhamnopyranosyl-(1→6)]-β-D-glucopyranoside (**5**) in a positive ionization mode

Quercetin 3-*O*-β-D-apiofuranosyl-(1→2)-[α-L-rhamnopyranosyl-(1→6)]-β-D-glucopyranoside (**5**) had the observed ion at *m/z* 765.1860 $[M+Na]^+$, calcd for $[C_{32}H_{38}O_{20}{}^{23}Na]^+$, 765.1849, $\Delta_{m/z}$ = 1.53 ppm, with molecular formula of $C_{32}H_{38}O_{20}$.

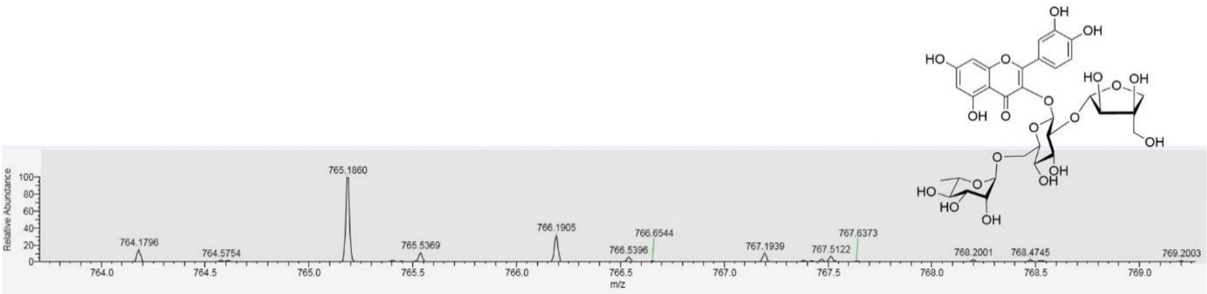

Figure S19. ^{1}H NMR (400 MHz) spectrum of kaempferol 3-*O*-β-D-apiofuranosyl-(1→2)-[α-L-rhamnopyranosyl-(1→6)]-β-D-glucopyranoside (**6**) in methanol-*d4*

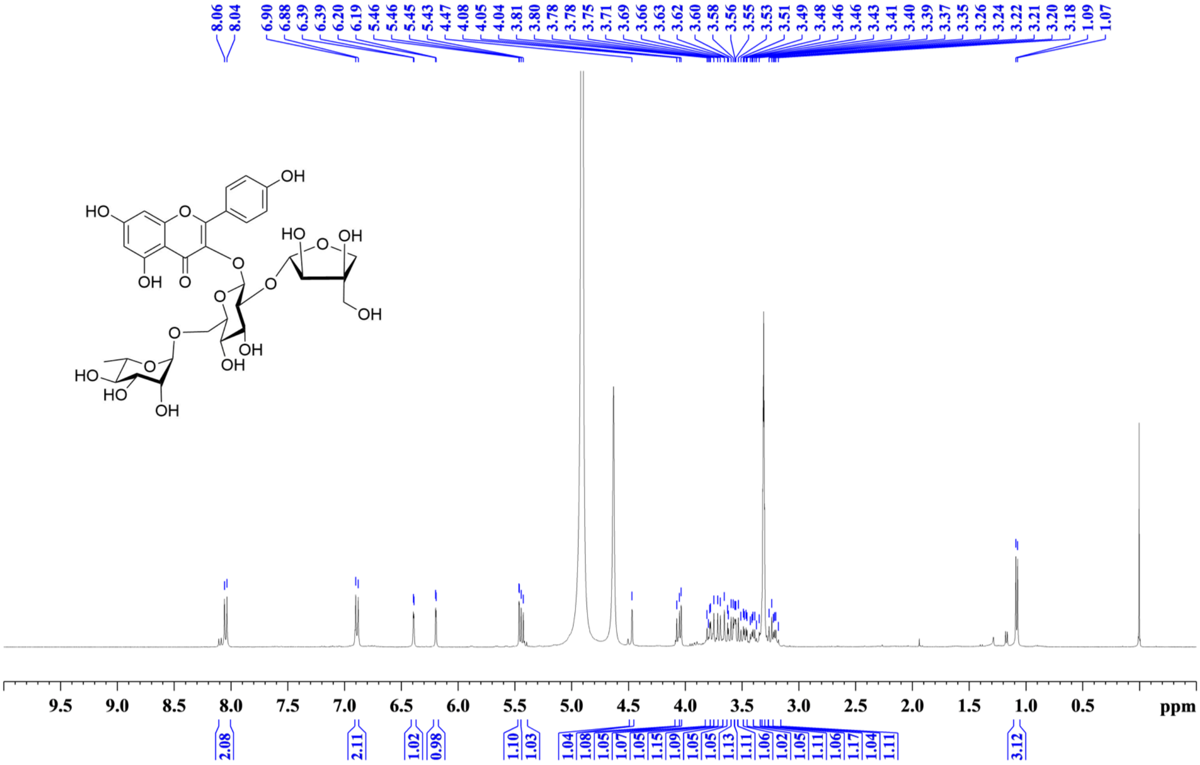

Figure S20. ^{13}C NMR (100 MHz) spectrum of kaempferol 3-*O*-β-D-apiofuranosyl-(1→2)-[α-L-rhamnopyranosyl-(1→6)]-β-D-glucopyranoside (**6**) in methanol-*d4*

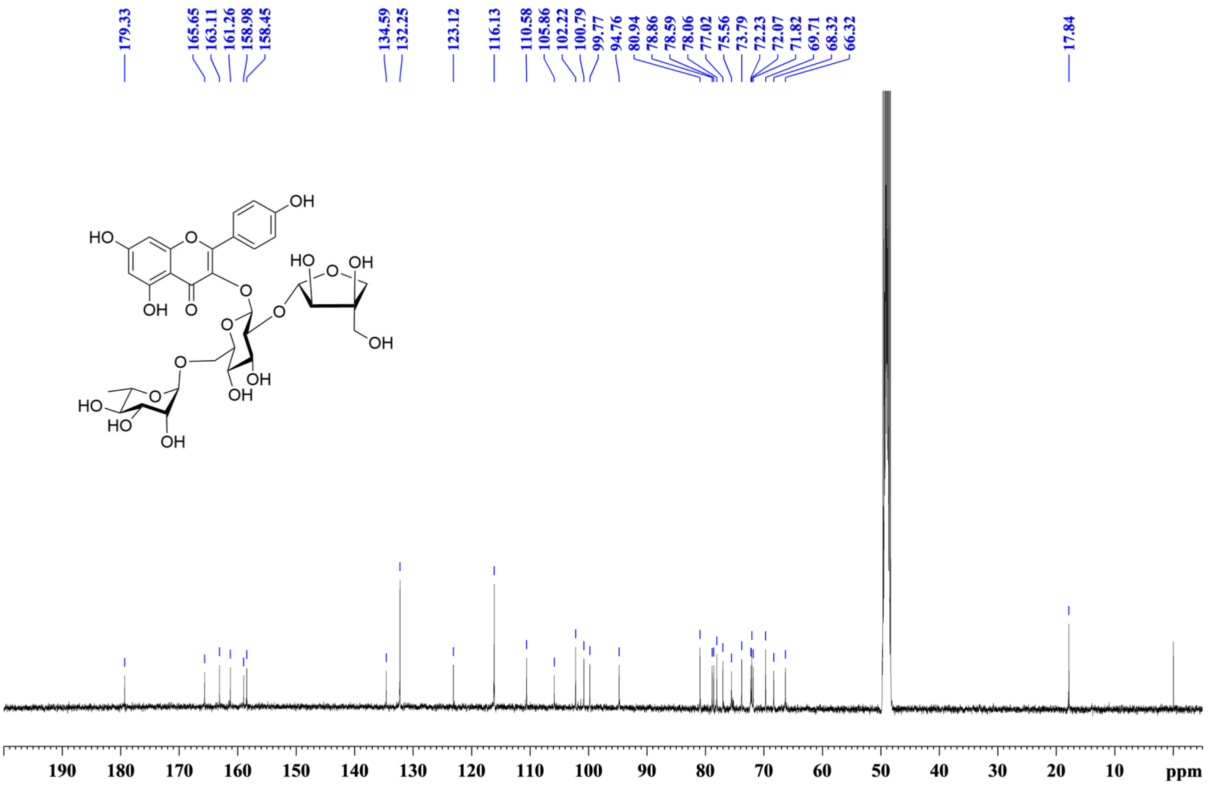

Figure S21. HRESI-MS spectrum of kaempferol 3-*O*-β-D-apiofuranosyl-(1→2)-[α-L-rhamnopyranosyl-(1→6)]-β-D-glucopyranoside (**6**) in a positive ionization mode

Kaempferol 3-*O*-β-D-apiofuranosyl-(1→2)-[α-L-rhamnopyranosyl-(1→6)]-β-D-glucopyranoside (**6**) had the observed ion at *m/z* 749.1913 [M+Na]$^+$, calcd for [$C_{32}H_{38}O_{19}{}^{23}Na$] 749.1899, $\Delta_{m/z}$ = 1.86 ppm, with molecular formula of $C_{32}H_{38}O_{19}$.

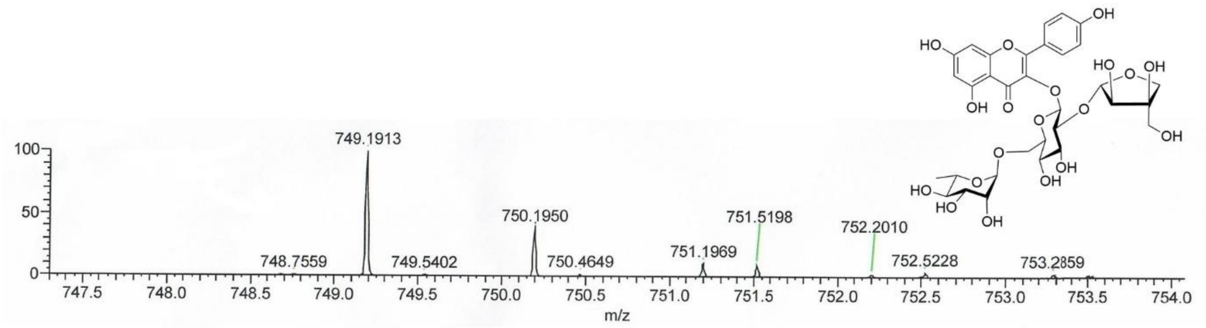

Figure S22. ^{1}H NMR (400 MHz) spectrum of kaempferol 3-*O*-β-D-apiofuranosyl-(1→2)-[α-L-rhamnopyranosyl-(1→6)]-β-D-galactopyranoside (**7**) in methanol-*d4*

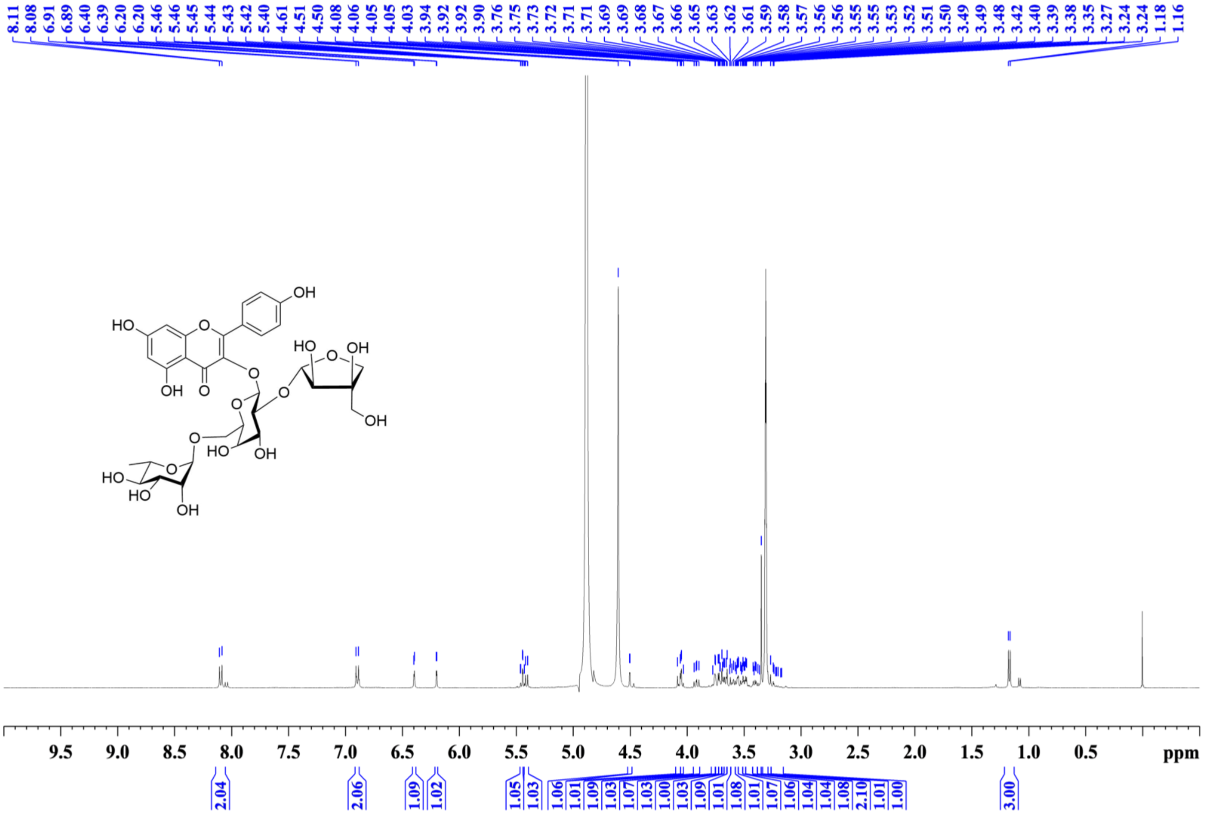

Figure S23. ^{13}C NMR (100 MHz) spectrum of kaempferol 3-*O*-β-D-apiofuranosyl-(1→2)-[α-L-rhamnopyranosyl-(1→6)]-β-D-galactopyranoside (**7**) in methanol-*d4*

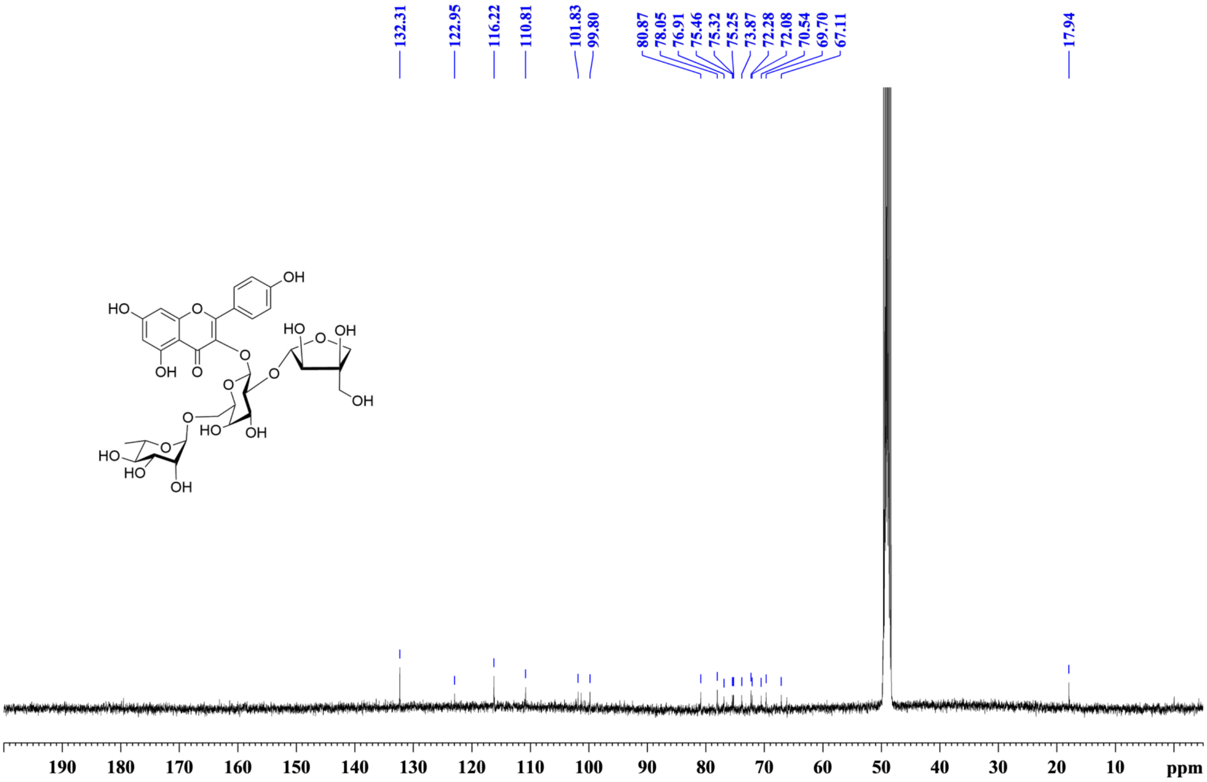

Figure S24. HSQC spectrum of kaempferol 3-*O*-β-D-apiofuranosyl-(1→2)-[α-L-rhamnopyranosyl-(1→6)]-β-D-galactopyranoside (**7**) in methanol-*d4*

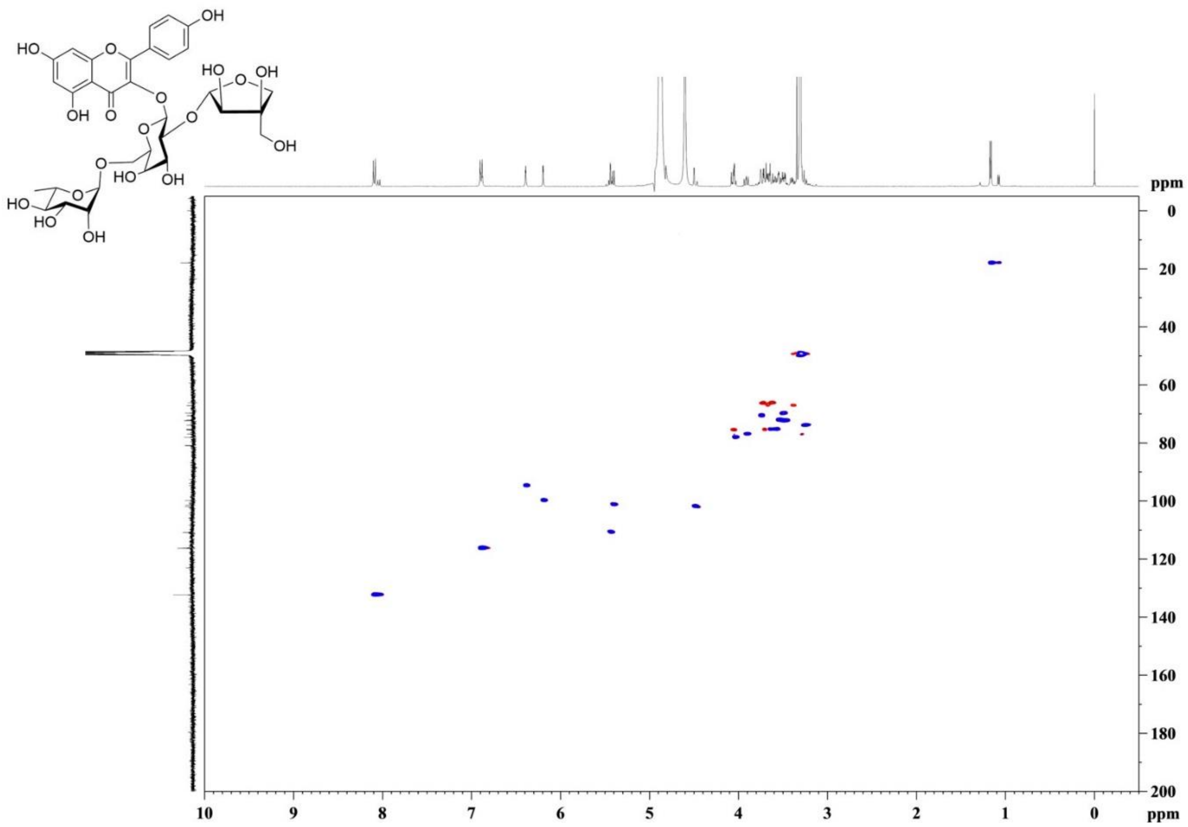

Figure S25. HMBC spectrum of kaempferol 3-*O*-β-D-apiofuranosyl-(1→2)-[α-L-rhamnopyranosyl-(1→6)]-β-D-galactopyranoside (**7**) in methanol-*d4*

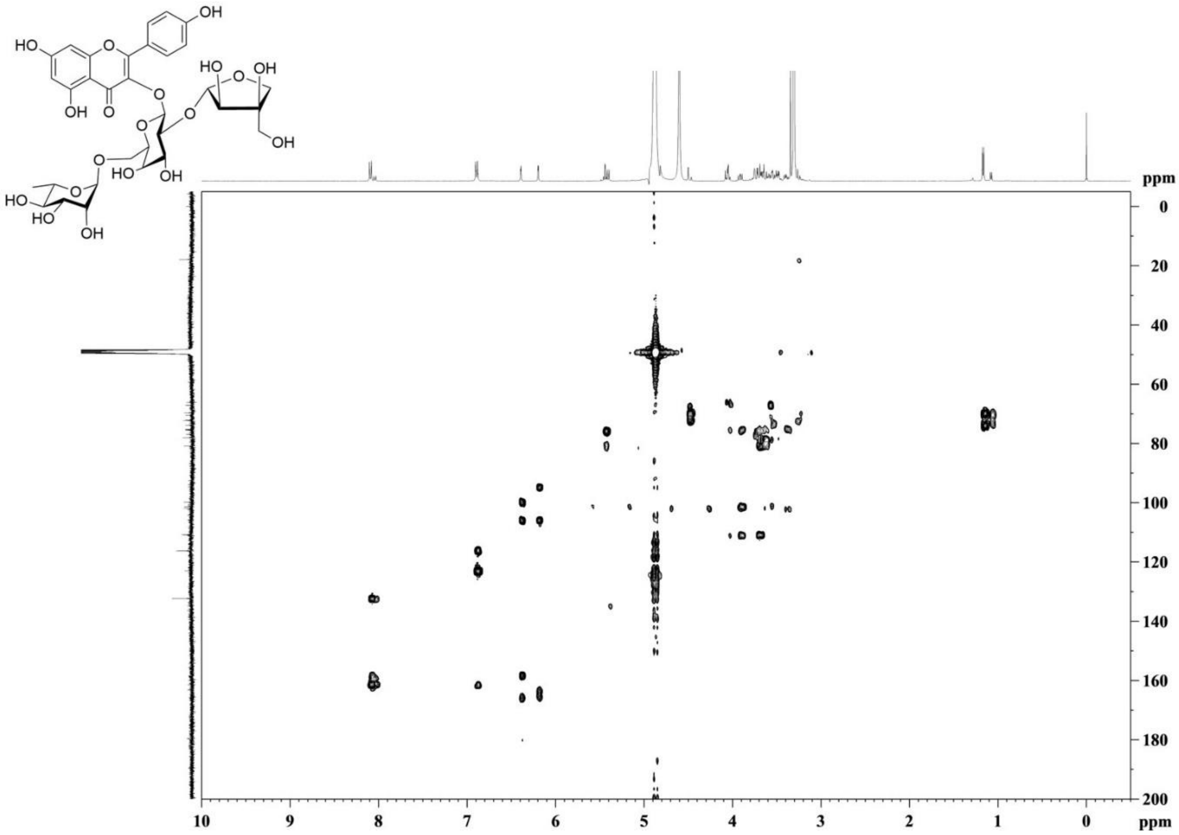

Figure S26. HRESI-MS spectrum of kaempferol-3-*O*-β-D-apiofuranosyl-(1→2)-[α-L-rhamnopyranosyl-(1→6)]-β-D-galactopyranoside (**7**) in a positive ionization mode

Kaempferol 3-*O*-β-D-apiofuranosyl-(1→2)-[α-L-rhamnopyranosyl-(1→6)]-β-D-galactopyranoside (**7**) had the observed ion at *m/z* 749.1901 $[M+Na]^+$, calcd for $[C_{32}H_{38}O_{19}{}^{23}Na]^+$, 749.1899, $\Delta_{m/z} = 0.15$ ppm, with molecular formula of $C_{32}H_{38}O_{19}$

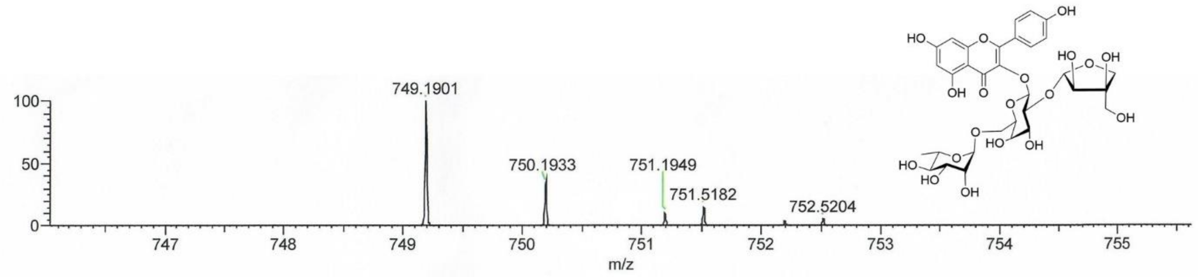

Figure S27. Dose-dependent inhibition of *C. grandis* crude extract towards α-glucosidase enzyme

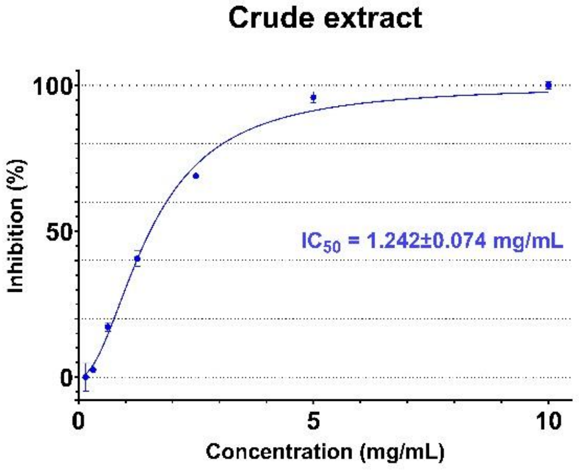

Table S1. ^{1}H (400 MHz) and ^{13}C NMR (100 MHz) data of compounds **1-4** in methanol-*d4*

	**Compound 1**		**Compound 2**		**Compound 3**		**Compound 4**			
**Position**	δ_C, type	δ_H, (*J* in Hz)	δ_C, type	δ_H, (*J* in Hz)	δ_C, type	δ_H, (*J* in Hz)	δ_C, type (**1**)	δ_H, (*J* in Hz)	δ_C, type	δ_H, (*J* in Hz)
**2**	159.5, C	-	159.4, C	-	159.3, C	-	159.3, C	-	159.0, C	-
**3**	135.8, C	-	135.5, C	-	135.7, C	-	135.6, C	-	135.8	-
**4**	179.6, C	-	179.4, C	-	179.6, C	-	179.4, C	-	179.4, C	-
**5**	163.1, C	-	163.0, C	-	163.0, C	-	163.3, C	-	163.3, C	-
**6**	100.1, CH	6.21, d (2.02)	99.9, CH	6.21, s	100, CH	6.22, d (1.72)	100.0, CH	6.21, d (2.04)	100.0, CH	6.21, d (2.04)
**7**	166.2, C	-	166.0, C	-	166.1, C	-	166.4, C	-	166.3, C	-
**8**	95.0, CH	6.40, d (2.02)	95.0, CH	6.41, s	94.9, CH	6.42, s	94.9, CH	6.40, dd (2.16)	94.9, CH	6.40, dd (2.16)
**9**	158.7, C	-	158.5, C	-	158.5, C	-	158.5, C	-	158.5, C	-
**10**	105.7, C	-	105.6, C	-	105.6, C	-	105.5, C	-	105.5, C	-
**1'**	123.2, C	-	122.7, C	-	122.6, C	-	123.1, C	-	122.8, C	-
**2'**	117.8, CH	7.67, d (2.09)	132.4, CH	8.06, d (8.64)	132.5, CH	8.09, d (8.76)	117.9, CH	7.88, d (2.12)	117.9, CH	7.67, d (2.08)
**3'**	146.0, C	-	116.1, CH	6.88, d (8.64)	116.1, CH	6.88, d (8.76)	145.8, C	-	145.7, C	-
**4'**	150.0, C	-	161.5, C	-	161.6, C	-	150.0, C	-	149.8, C	-
**5'**	116.2, CH	6.86, d (8.43)	116.1, CH	6.88, d (8.64)	116.1, CH	6.88, d (8.76)	116.1, CH	6.86, d (8.40)	116.0, CH	6.86, d (8.40)
**6'**	123.7, CH	7.62, dd (8.42, 2.10)	132.4, CH	8.06, d (8.64)	132.5, CH	8.09, d (8.76)	123.5, CH	7.62, dd (8.44, 2.12)	123.0, CH	7.59, dd (8.56, 2.20)
**1"**	104.8, CH	5.10, d (7.56)	104.6, CH	5.12, d (7.24)	105.5 CH	5.04, d (7.80)	104.7, CH	5.10, d (7.56)	106.0, CH	5.07, d (7.80)
**2"**	75.9, CH	3.33 – 3.50, m	75.7, CH	3.33 – 3.50, m	73.0, CH	3.71 – 3.82, m	75.7, CH	3.33 – 3.65, m	73.1, CH	3.73 – 3.83, m
**3"**	78.3, CH	3.33 – 3.50, m	78.1, CH	3.33 – 3.50, m	75.0, CH	3.48 – 3.65, m	78.2, CH	3.33 – 3.65, m	75.1, CH	3.33 – 3.65, m
**4"**	71.5, CH	3.25 – 3.30, m	71.4, CH	3.23 – 3.30, m	70.1, CH	3.71 – 3.82, m	71.4, CH	3.26 – 3.29, m	70.2, CH	3.73 – 3.83, m
**5"**	77.3, CH	3.33 – 3.50, m	77.2, CH	3.33 – 3.50, m	75.3, CH	3.48 – 3.65, m	77.2, CH	3.33 – 3.65, m	75.3, CH	3.33 – 3.65, m
**6"**	68.7, CH_2	3.80, d (10.34)<br>3.33 – 3.50, m	68.5, CH_2	3.79, d (10.28)<br>3.33 – 3.50, m	67.3, CH_2	3.71 – 3.82, m<br>3.35 – 3.40, m	68.5, CH_2	3.73 – 3.83, m<br>3.33 – 3.65, m	67.3, CH_2	3.73 – 3.83, m<br>3.33 – 3.65, m
**1"'**	102.6, CH	4.53, d (1.07)	102.4, CH	4.52, s	101.9, CH	4.52, s	102.4, CH	4.52, s	102.0, CH	4.52, s
**2"'**	72.2, CH	3.63, dd (3.24, 1.50)	72.1, CH	3.64, broad s	72.1, CH	3.48 – 3.65, m	72.1, CH	3.33 – 3.65, m	72.0, CH	3.33 – 3.65, m
**3"'**	72.3, CH	3.54, dd (9.49, 3.37)	72.3, CH	3.51, dd (9.48, 3.12)	72.3, CH	3.48 – 3.65, m	72.2, CH	3.33 – 3.65, m	72.2 CH	3.33 – 3.65, m
**4"'**	74.1, CH	3.25 – 3.30, m	73.9, CH	3.23 – 3.30, m	73.8, CH	3.25, d (9.48)	73.9, CH	3.26 – 3.29, m	73.8, CH	3.26 – 3.29, m
**5"'**	69.8, CH	3.33 – 3.50, m	69.7, CH	3.33 – 3.50, m	69.7, CH	3.48 – 3.65, m	69.7, CH	3.33 – 3.65, m	69.7, CH	3.33 – 3.65, m
**6"'**	18.0, CH_3	1.11, d (6.20)	18.0, CH_3	1.12, d (6.16)	18.0, CH_3	1.18, d (6.20)	18.0, CH_3	1.11, d (6.20)	17.9, CH_3	1.18, d (6.20)

Table S2. ^{1}H (400 MHz) and ^{13}C NMR (100 MHz) data of compounds **5-7** in methanol-*d4*

	Compound 5		Compound 6		Compound 7	
**Position**	δ_C, type	δ_H, (*J* in Hz)	δ_C, type	δ_H, (*J* in Hz)	$_C$, type	δ_H, (*J* in Hz)
**2**	158.9, C	-	159.0, C	-	[a]159.0, C	-
**3**	134.7, C	-	134.6, C	-	[a]135.2, C	-
**4**	179.3, C	-	179.3, C	-	[a]179.3, C	-
**5**	163.1, C	-	163.1, C	-	[a]163.8, C	-
**6**	99.7, CH	6.19, d (2.08)	99.8, CH	6.19, d (1.93)	99.8, CH	6.20, d (1.99)
**7**	165.7, C	-	165.6, C	-	[a]165.8, C	-
**8**	94.7, CH	6.38, d (2.05)	94.8, CH	6.39, d (1.87)	[a]94.8, CH	6.40, d (1.94)
**9**	158.4, C	-	158.4, C	-	[a]158.4, C	-
**10**	105.8, C	-	105.9, C	-	[a]105.9, C	-
**1’**	123.5, C	-	123.1, C	-	122.9, C	-
**2’**	117.3, CH	7.60, d (1.97)	132.2, CH	8.04, d (8.88)	132.3, CH	8.09, d (8.93)
**3’**	145.9, C	-	116.1, C	6.88, d (8.89)	116.2, C	6.89, d (8.92)
**4’**	149.6, C	-	161.3, C	-	[a]161.3, C	-
**5’**	116.1, CH	6.86, d (8.25)	116.1, CH	6.88, d (8.89)	116.2, CH	6.89, d (8.92)
**6’**	123.5, CH	7.62, dd (8.24, 2.13)	132.2, CH	8.04, d (8.88)	132.3, CH	8.09, d (8.93)
**1”**	100.9, CH	5.38, d (7.64)	100.8, CH	5.43, d (7.50)	101.8, CH	5.40, d (7.73)
**2”**	78.8, CH	3.62, dd (11.59, 7.49)	78.9, CH	3.58, m	76.9, CH	3.90, dd (7.76, 1.83)
**3”**	78.6, CH	3.51, dd (12.4, 9.01)	78.6, CH	3.52, d (9.10)	75.3, CH	3.48 – 3.63, m
**4”**	71.7, CH	3.22, dd (9.48, 2.62)	71.8, CH	3.22, d (9.18)	70.5, CH	3.64 – 3.75, m
**5”**	77.0, CH	3.27, t	77.0, CH	3.30, m	75.2, CH	3.48 – 3.63, m
**6”**	68.3, CH_2	3.78, d (9.50)	68.3, CH_2	3.80, dd (9.78, 5.08)	67.1, CH_2	3.64 – 3.75, m
		3.35, d (4.68)		3.30, m		3.39, dd (10.17, 3.46)
**1”’**	110.5, CH	5.47, d (7.38)	110.6, CH	5.46, d (7.64)	110.8, CH	5.44, d (6.83)
**2”’**	78.0, CH	4.05, d (1.11)	78.1, CH	4.04, s	78.0, CH	4.05. dd (7.85, 1.49)
**3”’**	80.9, C	-	80.7, C	-	80.9, C	-
**4”’**	75.6, CH_2	4.09, d (9.57)	75.6, CH_2	4.08, d (9.55)	75.5, CH_2	4.08, d (9.63)
		3.69, d (10.44)		3.69, s		3.64 – 3.75, m
**5”’**	66.3, CH_2	3.73, d (10.86)	66.3, CH_2	3.75, d (13.63)	66.2, CH_2	3.64 – 3.75, m
		3.62, dd (11.59, 7.49)		3.63, d (11.48)		3.48 – 3.63, m
**1””**	102.2, CH	4.48, d (1.38)	102.2, CH	4.47, s	[a]102.0, CH	4.51, s
**2””**	72.1, CH	3.57, dd (3.39, 1.68)	72.1, CH	3.58, m	72.1, CH	3.48 – 3.63, m
**3””**	72.2, CH	3.47, d (3.41)	72.2, CH	3.46, dd (9.45, 3.36)	72.3, CH	3.48 – 3.63, m
**4””**	73.8, CH	3.22, dd (9.48, 2.62)	73.8, CH	3.26, d (5.69)	73.9, CH	3.25, d (9.49)
**5””**	69.7, CH	3.41, dd (11.91, 5.82)	69.7, CH	3.39, m	69.7, CH	3.48 – 3.63, m
**6”’**	17.8, CH_3	1.09, d (6.13)	17.8, CH_3	1.07, d (6.21)	17.9, CH_3	1.16, d (6.21)

[a] *values were assigned from HSQC and HMBC spectra (Supplementary Figures S24 and S25)*
